# Novel Cinnamaldehyde Derivatives Inhibit Peripheral Nerve Degeneration by Targeting Schwann Cells

**DOI:** 10.3390/antiox11101846

**Published:** 2022-09-20

**Authors:** Yoo Lim Chun, Ki-Hoon Park, Badvel Pallavi, Won-Joon Eom, Chan Park, Youngbuhm Huh, Yeonjoo Lee, Jimin Lee, Sang Hoon Kim, Seung Geun Yeo, Hyung-Joo Chung, Byeong-Seon Kim, Na Young Jeong, Junyang Jung

**Affiliations:** 1 Department of Anatomy and Neurobiology, College of Medicine, Kyung Hee University, Dongdaemun-gu, Seoul 02447, Korea; 2 Department of Biomedical Science, Graduation School, Kyung Hee University, Dongdaemun-gu, Seoul 02447, Korea; 3 Department of Anatomy and Cell Biology, College of Medicine, Dong-A University, Seo-gu, Busan 49201, Korea; 4 Department of Anesthesiology and Pain Medicine, College of Medicine, Kosin University, Seo-gu, Busan 49267, Korea; 5 The Research Institute of Natural Science, Department of Chemistry Education, Gyeongsang National University, Gyeongnam, Jinju 52828, Korea; 6 Department of Otorhinolaryngology-Head and Neck Surgery, College of Medicine, Kyung Hee University, Dongdaemun-gu, Seoul 02447, Korea

**Keywords:** cinnamaldehyde, Schwann cells, peripheral nerve degeneration, TRPA1, Tg(mbp:eGFP) zebrafish model

## Abstract

Peripheral nerve degeneration (PND) is a preparative process for peripheral nerve regeneration and is regulated by Schwann cells, a unique glial cell in the peripheral nervous system. Dysregulated PND induces irreversible peripheral neurodegenerative diseases (e.g., diabetic peripheral neuropathy). To develop novel synthetic drugs for these diseases, we synthesized a set of new cinnamaldehyde (CAH) derivatives and evaluated their activities in vitro, ex vivo, and in vivo. The 12 CAH derivatives had phenyl or naphthyl groups with different substitution patterns on either side of the *α,β*-unsaturated ketone. Among them, **3f**, which had a naphthaldehyde group, was the most potent at inhibiting PND in vitro, ex vivo, and in vivo. To assess their interactions with transient receptor potential cation channel subfamily A member 1 (TRPA1) as a target of CAH, molecular docking studies were performed. Hydrophobic interactions had the highest binding affinity. To evaluate the underlying pharmacological mechanism, we performed bioinformatics analysis of the effect of **3f** on PND based on coding genes and miRNAs regulated by CAH, suggesting that **3f** affects oxidative stress in Schwann cells. The results show **3f** to be a potential lead compound for the development of novel synthetic drugs for the treatment of peripheral neurodegenerative diseases.

## 1. Introduction

Peripheral nerve degeneration (PND) is essential after nerve injury because nerve degeneration determines efficient peripheral nerve regeneration. Anterograde peripheral nerve degeneration, also known as Wallerian degeneration, is linked to axon degradation, demyelination, Schwann cell dedifferentiation and proliferation, and macrophage recruitment. These are regulated by Schwann cells, unique peripheral neuroglial cells [1]. However, during PND, systemic nerve damage, such as hyperglycemic conditions, decreases the regenerative capacity of Schwann cells, which undergo irreversible nerve degeneration. This leads to irreversible peripheral neurodegenerative diseases (e.g., diabetic peripheral neuropathy). At present, such diseases are treatable only palliatively, such as with pain control [2].

Cinnamaldehyde (CAH; 3-phenyl-2-propenal) is an oily yellow liquid with a cinnamon odor that is naturally synthesized by the seven-step shikimate pathway in several plants of the genus *Cinnamomum* [3,4]. CAH has antifungal, antibacterial, antitumor, anti-inflammatory, and cardioprotective activities [5]. Additionally, CAH exerts a protective effect on brain injury, neuroinflammation, and Parkinson’s disease [6,7,8]. In a number of previous medicinal applications, CAH derivatives had anti-diabetic, neuroprotective, and antioxidant effects [9,10]. These studies indicated that CAH might be a potent scaffold for presenting bioactivity, and its medicinal properties can induce new pharmacological effects. However, CAH has not been studied for its effect on PND and Schwann cells.

In this study, we investigated the medicinal applications of synthetic derivatives based on CAH as a basic scaffold because CAH can have limited clinical application by causing allergic reactions [4]. Using in vitro-dedifferentiated Schwann cells, ex vivo PND models, and in vivo transgenic zebrafish models, we evaluated the effects of CAH derivatives on PND. Moreover, in vivo zebrafish toxicity assays suggested directions for lead optimization to reduce the in vivo toxicity of the CAH derivatives. In previous studies, transient receptor potential cation channel subfamily A member 1 (TRPA1) was determined as a target of CAH [11,12]. After peripheral nerve injury, TRPA1 is upregulated and controls neuroinflammation in Schwann cells [13]. Because TRPA1 enhances macrophage infiltration into the injured peripheral nerves [14], TRPA1 could increase its myelin engulfment during PND [15]. Thus, we performed molecular docking analysis between CAH derivatives and TRPA1 as a potential target of CAH derivatives.

## 2. Materials and Methods

### 2.1. Chemicals and Instruments

All chemicals were purchased from Acros (Geel, Belgium), Alfa Aeser (Haverhill, MA, USA), Fisher (Waltham, MA, USA), and Sigma-Aldrich (St. Louis, MO, USA) and solvents were obtained from Daejung (Siheung, Korea) and Damul Chem (Gunpo, Korea), used as received without further purification. All reactions were conducted under an air atmosphere. All vials and glassware were dried in an oven. Organic solutions were concentrated under reduced pressure on a rotary evaporator using a water bath. Thin-layer chromatography was performed on Whatman precoated silica gel 60 F-254 plates (Supelco, Bellefonte, PA, USA) and visualized by ultraviolet light. Flash chromatography was performed with Kanto Silica gel 60 N (40–50 mesh, Tokyo, Japan). NMR spectra were obtained using a Brüker 300 MHz Fourier-transform NMR spectrometer (Brüker, Billerica, MA, USA) at the Gyeongsang National University. ^1^H and ^13^C chemical shifts in parts per million (δ) were referenced to internal tetramethylsilane (TMS).

### 2.2. General Procedure

**Figure 1:**
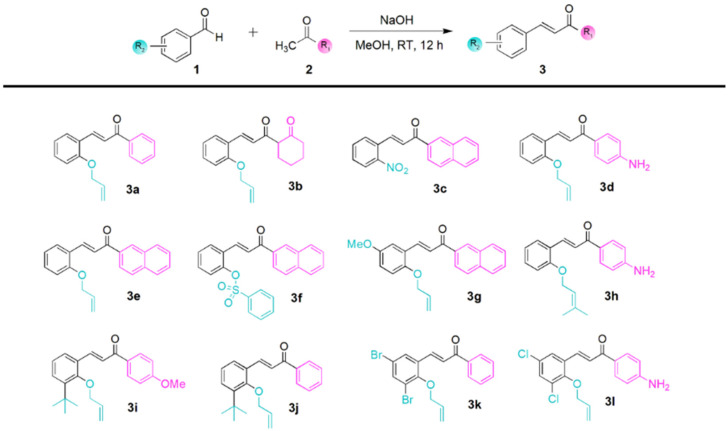
Synthesis of CAH derivatives (*α,β*-unsaturated ketones) from Claisen–Schmidt condensation of oxygenated acetophenones. Substrate scope and structures of CAH derivatives (**3a**–**3l**) are shown in the lower panel.

An oven-dried 25 mL vial equipped with a stir bar was charged with an aldehyde **1** (2.0 mmol, 1.0 equiv.) and a ketone **2** (2.0 mmol, 1.0 equiv.) in methanol (5 mL) (Figure 1). The reaction mixture was stirred for 5 min until both starting materials were dissolved. NaOH (3.3 mmol, 1.1 equiv.) was dissolved in a methanol–water mixture (1:1, 10 mL). NaOH was added to the vial in the methanol–water mixture. Then, the reaction mixture was stirred at room temperature (RT) for 12 h until TLC showed complete consumption of aldehyde and ketone. The reaction mixture was diluted with EtOAc (15 mL) and quenched with ice water (15 mL). The organic layer was separated, and the aqueous solution was extracted twice with EtOAc (15 mL). The combined organic layer was washed with brine (20 mL), dried over MgSO_4_, filtered, and the solvent was removed under reduced pressure. The residue was purified by flash column chromatography on silica gel to afford the desired products **3** [16,17].

### 2.3. Cell Culture

The mouse neuronal Schwann cell line, SW10 (CRL-2766), and the human Schwann cell line, hTERT ipn02.3 2λ (hTERT02.3, CRL-3392), were purchased from American Type Culture Collection (ATCC, Manassas, VA, USA). SW10 cells were then maintained at subconfluence in Dulbecco Modified Eagle’s Medium (DMEM, SH30243.01, Cytiva, Marlborough, MA, USA) supplemented with 1% (*v/v*) penicillin/streptomycin (30-022-CI, Corning, NY, USA) and 9% (*v/v*) fetal bovine serum (FBS, SH30919.03, Cytiva, Marlborough, MA, USA) in an incubator at 33 °C under a humidified atmosphere of 5% CO_2_. hTERT02.3 cells were incubated in DMEM added with 1% (*v/v*) penicillin/streptomycin, 9% (*v/v*) FBS, and 2 mM L-glutamine (25030-081, Gibco, Waltham, MA, USA) at 37 °C.

### 2.4. Cell Viability Assay

To determine the anti-proliferative effect of compounds on SW10 cells, cell viability was assessed by MTS assay using the Cell Titer 96 AQueous One Solution Cell Proliferation Assay kit (Promega, G3580, Madison, WI, USA) according to the manufacturer’s instructions. Briefly, a 100 µL culture medium containing 1.92 × 10^4^ SW10 cells was distributed equally in each well of 96-well plates with the presence of compounds (1 µM, 10 µM, and 100 µM) and incubated for 9 h. At the designated incubation time, 20 µL MTS solution reagent were added to each well evenly and reacted for 1 h. After the reaction, optical density at 492 nm was determined to examine cell viability using a microplate spectrophotometer (51119000, Thermo Fisher Scientific, Waltham, MA, USA).

### 2.5. Trypan Blue Exclusion Assay

SW10 cells were plated in a 6-well plate under the same condition as for the cell viability assay. After 10 h treated with compounds in concentrations of 1 µM, 10 µM, and 100 µM, SW10 cells were trypsinized. The cell suspension was then centrifuged at 300× *g* for 5 min at RT and the supernatant was removed. The cell pellet was resuspended by 1 mL of DMEM, and 10 µL of the cell suspension were stained with 10 µL of trypan blue solution (0.4% (*w/v*), 15250-061, Thermo Fisher Scientific, Waltham, MA, USA). Counts of live and dead cells were determined visually using a hemocytometer and light microscope. The ratio of live cells and dead cells was defined as cell death (%).

### 2.6. Drug Efficacy Indices

The percentage of cell viability (%) was estimated as (absorbance of treated cells − absorbance of background)/(absorbance of matched controls − absorbance of background controls) × 100 (%). Normalized growth rate inhibition (%) was evaluated by means of absorbance (nm) of untreated cells at the time of compound treatment. After calculating the average of both live and dead cells, the percentage of cell death (%) at each concentration was assessed. The half-maximal inhibitory concentration (IC_50_), the half-maximal growth inhibition (GI_50_), and the half-maximal effective concentration (EC_50_) were determined by linear regression analysis [18,19].

### 2.7. Molecular Docking

Three-dimensional structures of transient receptor potential ankyrin 1 (TRPA1), ATP binding cassette subfamily G member 2 (ABCG2), P-glycoprotein (P-gp), 5α-reductase, androgen receptor (AR), Sirtuin1 (SIRT1), p53, and histone deacetylase (HDAC) were obtained from the protein data bank (PDB, https://www.rcsb.org/; accessed on 10 March 2022). Three-dimensional structures of the compounds used for ligands were modeled via Chem3D Ultra (version 8.0; CambridgeSoft, Cambridge, MA, USA). First, the AutoDock Tool (version 4.2.6, The Scripps Research Institute, La Jolla, CA, USA) was used to delete all water molecules from the protein, allot hydrogen polarities, and add the Kollman charges and Gasteiger charges. AutoDock Vina was also used for docking to estimate the interaction energies between the protein and the ligand. The results were analyzed by Discovery Studio Visualizer (version 21.1.0; BIOVIA Dassault Systèmes, San Diego, CA, USA).

### 2.8. Animals

Male C57BL/6 mice, 5 weeks old, and 20–22 g each, purchased from Orientbio™ (Seongnam, Korea), were used in this study. Mice were raised under a condition with a 12 h light/12 h dark cycle, constant temperature of 23 ± 1 °C, and 50% humidity. Additionally, all experimental animals were provided with sufficient feeds and water. All experimental animal procedures were proceeded according to the protocol authorized by Kyung Hee University Committee of Animal Research [KHSASP-21-463] to minimize the number and the suffering of all experimental animals. Including sacrifices, all animal experiments were performed in agreement with the institutional guidelines of the Korean Academy of Medical Science.

### 2.9. Sciatic Nerve Explant Culture

Fresh sciatic nerve explant was performed according to a previous protocol [20]. In short, sciatic nerves enveloped with connective tissue were collected from the mice using a fine iris scissor (FST, Foster City, CA, USA) without any damage. Tissues were placed in cold phosphate-buffered saline (PBS) under a stereomicroscope to remove connective tissues and cut into fragments of 3 to 4 mm long by fine iris scissors. Fragmented pieces of the sciatic nerves were placed in a fresh culture medium with or without compounds. Explants were incubated at an incubator of 37 °C and 5% CO_2_ condition for 3 days. After treatment, the explants were washed with 1 × PBS and relocated to 4% paraformaldehyde (PFA) for fixation.

### 2.10. Immunohistochemistry

Immunohistochemistry was progressed in a previous study [20]. Briefly, teased sciatic nerves mounted on slides underwent post-fixation with 4% PFA at RT and washed with 1 × PBS for further immunostaining. The slides were then permeabilized using 0.3% Triton X-100-added PBS (PBS-T; T1020, Biosesang, Seongnam, Korea) at RT, followed by washing with 1 × PBS. For blocking nonspecific binding, we used 5% of FBS and 5% of bovine serum albumin (BSA; Bovogen Biologicals, Keilor East, Australia) overnight at 4 °C and subsequently incubated them with primary antibodies. To designate sciatic nerve degeneration, the following antibodies were used: neurofilament (NF, 1:1000, BioLegend, San Diego, CA, USA), S100-β (S100; 1:1000, s2532, Sigma Aldrich, St. Louis, MO, USA), myelin basic protein (MBP; 1:1000, ab980, Millipore, Darmstadt, Germany), lysosomal-associated membrane protein 1 (LAMP1; 1:1000, sc-19992, Santa Cruz Biotechnology, Dallas, TX, USA), and cyclin D1 (CCND1; 1:1000, MA5-14512, Invitrogen, Carlsbad, CA, USA). Unbound primary antibodies were washed 3 times with 1 × PBS and then reacted with Alexa Fluor conjugated secondary antibodies (Alexa Flour-488 Donkey anti-rabbit or anti-rat and Alexa Flour-594 Donkey anti-rabbit or anti-mouse, Invitrogen, Carlsbad, CA, USA), under a light protected environment at RT for 2 h. Excess secondary antibodies were washed with 1 × PBS 3 times, and nuclei were counterstained with 4′,6-Diamidine-2′-phenylindole dihydrochloride (DAPI; 1:1000, 10236276001, Roche, Basel, Switzerland) at RT for 1 min. Finally, the slides were mounted with a Gel mount (Biomeda, Burlingame, CA, USA) for imaging by laser scanning confocal microscope (LSM710, Carl Zeiss, Oberkochen, Germany).

### 2.11. Morphometric Indices

Sciatic nerves were screened for morphological phenotypes to quantitatively determine peripheral nerve degeneration. The number of transverse stripes (bands of Fontana) in 2 mm-long sciatic nerves monitored under an Axiophot upright microscope installed with differential interference contrast (DIC) filters (Carl Zeiss, Oberkochen, Germany) was defined as the stripe index. Ovoid-shaped myelin, representing myelin fragmentation, was measured by counting the number of ovoids in 200 µm-long teased sciatic nerve fibers. Myelin index was selected to assess the myelin sheath quantitatively in immunostained sciatic nerve fibers, referring to the number of consecutive MBP immunostained lines. For quantitative measurement of intact axons, the NF index was selected, meaning the consecutive immunostained lines of NF. Immunostained consecutive lines used in both Myelin and NF indices were selected over 100 µm-long sciatic nerve fibers among a total of 100 sciatic nerve fibers.

### 2.12. Bioinformatics and Validation

The differentially expressed (DE) genes (DEGs) and DE miRNAs expression profiles were collected from non-small cell lung cancer (NSCLC) cell lines (SK-MES-1 and NCI-H226 cell lines) via whole transcriptome sequencing [21]. Briefly, genes or miRNA with a Fragments Per Kilobase of transcript per Million (FPKM) of each sample > 1, the absolute value of |log_2_ (Fold Change)| > 1, and adjusted *p*-value < 0.05 were considered as DEGs and DE miRNAs. The DEGs and DE miRNAs were evaluated by hTERT02.3 cells treated with **3f** for 5 h.

#### 2.12.1. Functional Enrichment Analysis

To explore the biological function of selected DEGs and DE miRNAs, gene ontology (GO) and pathway enrichment analyses were performed on The Database for Annotation, Visualization, and Integrated Discovery (DAVID) bioinformatics resources (2021) [22]. Significant results of the GO biological process (BP) and Kyoto Encyclopedia of Genes and Genomes (KEGG) with a cut-off of adjusted critical criterion < 0.05 were selected.

#### 2.12.2. Quantitative Real-Time Polymerase Chain Reaction (qPCR)

To perform qPCR for validating DEGs and DE miRNAs in Schwann cells, the total RNA of hTERT02.3 cells was isolated using the Tri-Reagent method (Molecular research center, Cincinnati, OH, USA), according to the manufacturer’s instructions. Total RNA (3 µg) was then reverse-transcribed to cDNA using 1 µL of 0.1 M Dithiothreitol (DTT), 4 µL of 5 × first-strand buffer, and 0.3 µg of oligo dT via Superscript reverse III transcriptase kit (18080-044, Invitrogen, Carlsbad, CA, USA). To detect mature microRNA (miRNA) expression, the Tri-Reagent method was also used to isolate total RNA and then amplified by the Quantimir method (System Biosciences, RA420A-1, Palo Alto, CA, USA). Afterward, qPCR was proceeded with 2 × SYBR Green (Takara Bio Co., Otsu, Japan) using Thermal Cycler Dice Real Time System Lite (Takara Bio Co., Otsu, Japan). Each Cq data of genes and miRNAs was exported to Microsoft Excel for further evaluation. Each gene or miRNA had three technical replicates leading to generating three individual Cq values. The mean of the triplicates of every gene or miRNA was calculated to be the representative Cq value of the biological sample. The average of human GAPDH and U6 was used as an internal control for mRNA and miRNA, respectively. Primer sequences for validation are listed in Table S1.

### 2.13. Zebrafish Husbandry

The care and maintenance of zebrafish were in accordance with the guidelines and regulations of the Kyung Hee University Committee on Animal Research with approval (Protocol number #KHSASP-21-302). The transgenic fish line [Tg(mbp:eGFP)] was provided by the Zebrafish Center for Disease Modeling (ZCDM, Daejeon, Korea). Adult zebrafish were kept at 28.5°C under a 14:10 h (light:dark) photoperiod. The fish were fed twice a day with brine shrimp and commercial food [Tetramin^®^ Tropical Flakes (#77101, Blacksburg, VA, USA)]. To obtain embryos, an egg trap was placed overnight in a tank containing male and female specimens (2:1 ratio) one day prior to testing. One hour after the beginning of the light cycle, eggs were collected with a Pasteur pipette and rinsed with egg water (0.3 mg of sea salt/L). Viable fertilized eggs were selected for embryo toxicity assays.

### 2.14. Exposure of Zebrafish Embryos to **3f**

Zebrafish embryos (4 h post-fertilization) were exposed to **3f** (final 0.01 to 10 µM). After waterborne exposure of embryos to a solution containing **3f** for 120 h, live embryos were observed under an SMZ800 stereomicroscope and photographed using a DIGITAL SIGHT DS-Fi3 (Nikon, Tokyo, Japan) every 24 h. In brief, three sets of 25 embryos per each set were morphologically evaluated for up to 120 h during the method based on the criteria in the previous report [23]. Larvae with no movement to the touch were considered dead. The number of deaths (lethal endpoints) and sublethal effects were used to calculate the LC_50_.

### 2.15. Statistical Analysis

The results appeared as means and standard deviations. The differences between experimental groups were analyzed using the Student’s *t*-test through IBM SPSS Statistics 2.3 (Armonk, North Castle, NY, USA). * *p* < 0.05, ** *p* < 0.01, and *** *p* < 0.001 were regarded as statistically significant.

## 3. Results and Discussion

### 3.1. Synthesis of CAH Derivative 3

We synthesized 12 CAH derivatives (*α, β*-unsaturated ketone derivatives, **3a**–**3l**) by the Claisen–Schmidt condensation of oxygenated acetophenones with benzaldehyde derivatives, affording a series of alkylated CAH derivatives (**3a**–**3l**) in the presence of NaOH using methanol as a solvent in moderate to high yields [16,17]. The CAH derivatives were characterized by ^1^H NMR and ^13^C mass spectral analysis. The synthetic procedure of CAH derivatives and their structures are described in Figure 1. Among **3a**–**3l**, some CAH derivatives showed similar structures to chalcones (CL, 1,3-diaryl-2-propen-1-ones), a subclass of structural analogs of flavonoids. CLs reportedly exhibit diverse biological activities, including antibiotic and anticancer activities mediated by the enone and 2′-hydroxy groups. However, because the compounds contained groups other than CLs, we used the term CAH derivatives for our newly synthesized *α,* *β*-unsaturated ketones. Their synthesis is described in the Supplementary Materials.

### 3.2. In Vitro Assay

Stopping or delaying PND is important because peripheral nerves irreversibly lose their regenerative potency in the absence of functioning Schwann cells. In vitro drug efficacy can be evaluated by calculating therapeutic indices such as the half-maximal inhibitory concentration (IC_50_). However, because Schwann cell proliferation is a physiological event, unlike cancer cells, cytotoxicity-related indices such as the IC_50_ or half-maximal effective concentration (EC_50_) are unsuitable for quantifying Schwann cell proliferation [20]. Therefore, we calculated the half-maximal growth inhibition concentration (GI_50_) based on cell viability assays. IC_50_ and EC_50_ values were used as supplementary indices to assess the toxicity of the synthetic compounds.

**Figure 2:**
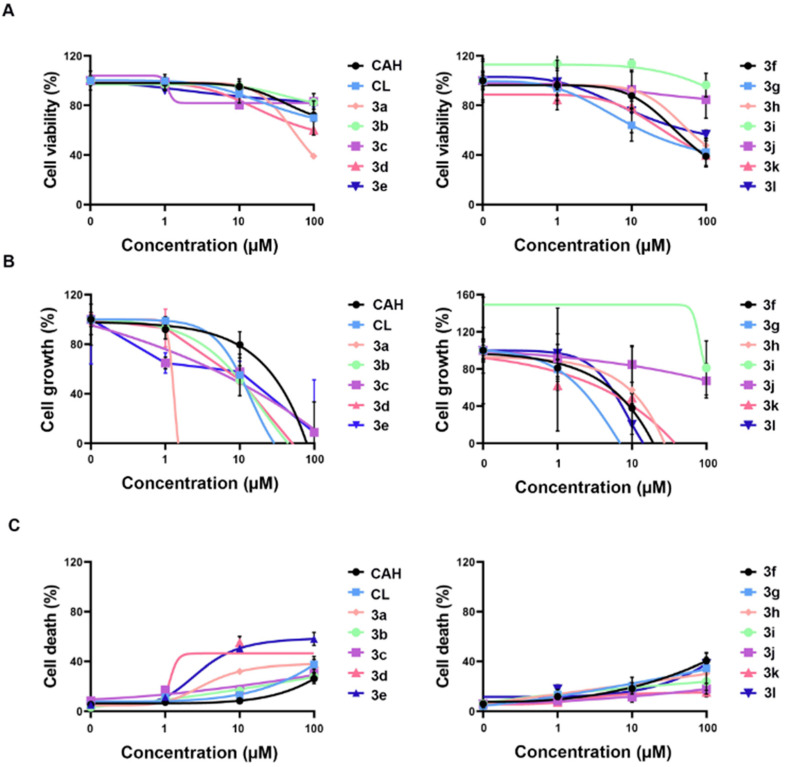
In vitro efficacy of CAH derivatives **3a**–**3l** using therapeutic indices in SW10 cells treated with 1–100 µM of each compound for 10 h. (**A**) Dose–effect curve plot of **3a**–**3l** indicated the cell viability of SW10 cells. (**B**) Cell growth curves of **3a**–**3l** representing inhibition of cell proliferation were determined by the MTS assay in a dose-dependent manner. (**C**) Cell death was calculated as a ratio of live cells over dead cells via trypan blue exclusion assay. All CAH derivatives showed no in vitro efficacy in concentrations of ≤100 nM and 100% cell death in the concentration of 1 mM, respectively. CAH, cinnamaldehyde. CL, chalcone.

We performed cell viability assays using CAH and CL as positive controls and an SW10 cell line as an in vitro-dedifferentiated Schwann cell model because Schwann cell dedifferentiation occurs during PND [20]. Among the derivatives, **3a** showed a marked inhibitory effect on Schwann cell proliferation. CAH, CL, **3d**, **3f**, **3g**, **3h**, and **3l** showed moderate inhibitory effects (Figure 2A). In a growth inhibition (%) assay, CAH, CL, **3a**, **3e**, **3f**, **3h**, and **3k** inhibited growth, whereas **3a** and **3g** showed an inverse growth effect (i.e., they were cytotoxic; Figure 2B). These results are in agreement with those of a trypan blue exclusion assay. Overall, **3d** and **3e** showed higher cell death rates compared to other derivatives (Figure 2C). In vitro, **3f**, **3h**, and **3k** targeted Schwann cells to inhibit PND without cytotoxicity. The therapeutic indices of the compounds are listed in Figure S1.

### 3.3. Molecular Docking Studies

TRPA1 is a transient receptor potential cation channel that functions as a chemical stress sensor and is activated by isothiocyanate and CAH [24]. TRPA1 in Schwann cells mediates pain signals after nerve injury [13]. TRPA1 is also involved in macrophage infiltration, which increases neuroinflammation and myelin engulfment during PND [14,15]. Furthermore, because TRP melastatin 7 (TRPM7), like other TRP families, is also known to inhibit PND [25], TRPA1 may have the potential as a therapeutic target in Schwann cells for PND. To select one compound for further evaluation, we set up molecular docking between **3a**–**3l** and TRPA1 as a filter to reduce the candidates and evaluated their binding affinity compared to that of CAH as the control because CAH is a potential TRPA1 ligand [26,27].

**Figure 3:**
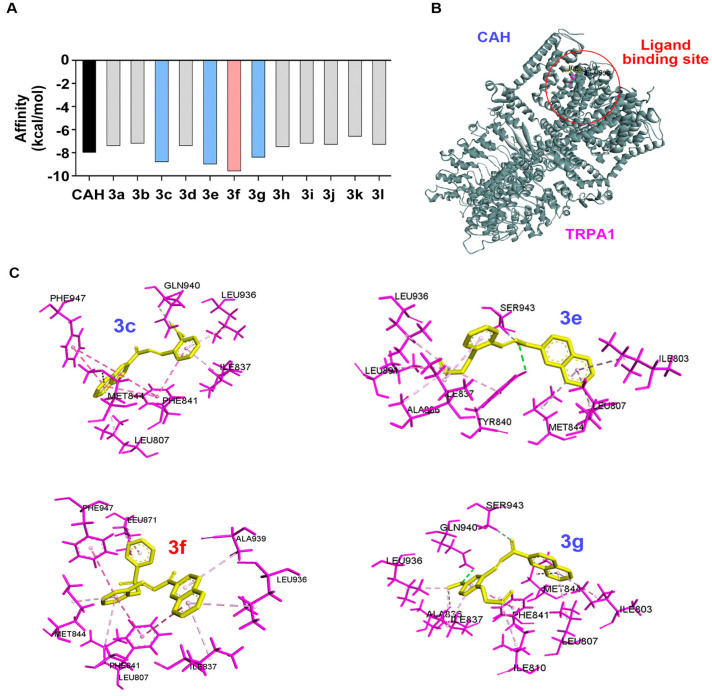
Docking-simulation results between TRPA1 and CAH derivatives. (**A**) The binding affinity energies (kcal/mol) of **3a**−**3l** with TRPA1 were estimated by AutoDock Vina software. (**B**) Three-dimensional structure of TRPA1 (PDB code: 6X2J) was used from the Protein Data Bank database (https://www.rcsb.org/; accessed on 10 March 2022). (**C**) Docking conformations of **3c**, **3e**, **3f**, and **3g** showing high affinity with TRPA1 were visualized, and the zoom-in view of each docked ligand inside the TRPA1 was displayed. The structure of ligands was represented as yellow, the residues in the ligands were indicated as pink, and predicted hot spots were shown as blue. CAH, cinnamaldehyde.

The binding free energy between TRPA1 and the 12 derivatives is shown in Figure 3 and Table S2. CAH derivatives with naphthaldehyde (**3c**, **3e**, **3f**, and **3g**) had a greater affinity (−8.8, −9.0, −9.6, and −8.4 kcal/mol, respectively) for TRPA1 than CAH (−8.0 kcal/mol) (Figure 3A). The remaining CAH derivatives with a vinylphenyl or phenyl group were predicted to have a lower affinity. Furthermore, **3c**, **3e**, **3f**, and **3g** had a naphthyl group as the substituent, not a phenyl group, suggesting that large aromatic groups favor interactions with the binding site. We next evaluated interaction profiles (Figure 3B) using the top three most stable CAH derivatives, including naphthaldehyde (**3c**, **3e**, and **3f**) with TRPA1 using AutoDock Vina [28]. π–π interactions (πs) and π-alkyl interactions (πa) were observed in **3c** (πs: Phe841 and Phe947; πa: Leu807, Ile837, and Leu936), **3e** (πa: Ile803, Leu807, Ala836, Ile937, Met844, Leu891, and Leu936), and **3f** (πs: Phe841 and Phe947; πa: Leu807, Ile837, Met844, Leu871, Leu936, and Ala939). Details of the interaction profiles of the four compounds are shown in Figure 3C.

We also performed molecular docking between **3f**, which bound TRPA1 with the highest stability, and protein targets of CL [29]. ABCG2, P-gp, 5α-reductase, AR, SIRT1, p53, and HDAC were used for molecular docking [30]. The target proteins, except p53, showed a high binding affinity with **3f** (Figure S2). P-gp, 5α-reductase, SIRT1, and HDAC are expressed in Schwann cells and are involved in Schwann cell myelination [31,32,33,34]. Therefore, CAH derivatives with high binding affinities for TRPA1 docked with a hydrophobic region of TRPA1, and CAH derivatives with a naphthyl group had more stable interactions with the TRPA1binding site. Additionally, **3f** may have multiple targets such as P-gp, 5α-reductase, SIRT1, and HDAC, as well as TRPA1 during PND.

### 3.4. Ex Vivo and In Vivo Efficacy Assays

**Figure 4:**
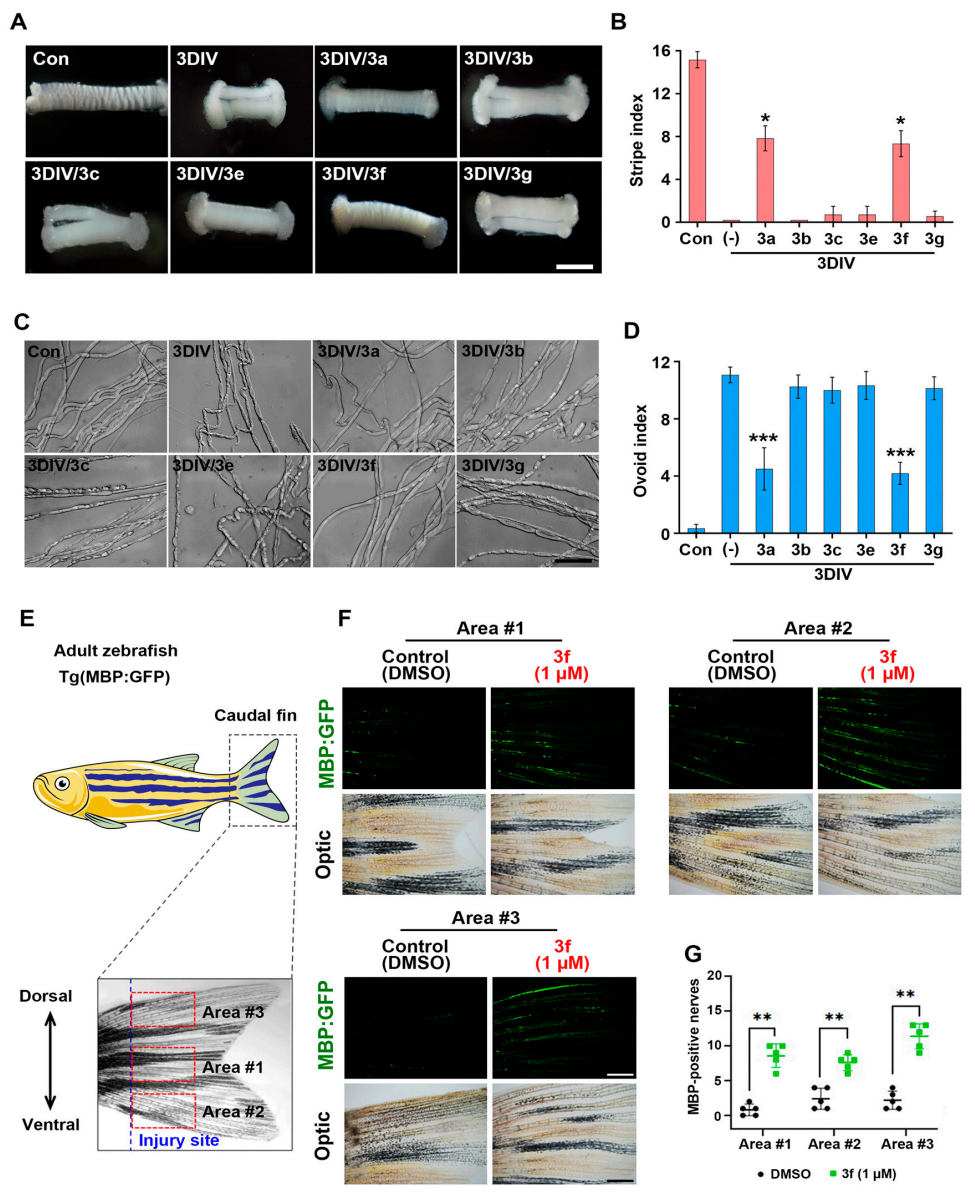
Ex vivo and in vivo efficacy of CAH derivatives against PND. (**A**) Sciatic nerve explant culture was used for evaluating ex vivo efficacy. Transverse stripes of sciatic nerve explants in the presence or absence of compounds of 100 µM were observed and photographed under a stereomicroscope for three days in vitro (3DIV). Scale bar, 50 µm. The compounds were **3a**, **3b**, **3c**, **3e**, **3f**, and **3g**. (**B**) The number of transverse stripes in a 2 mm-long sciatic nerve was defined as stripe index and measured by counting under a stereomicroscope (n = 3 mice). (**C**) Ovoid-shaped myelin formation appeared in the sciatic nerve fibers cultured for 3DIV with or without treatment of the compounds. (**D**) The ovoid index was obtained by counting the ovoid-shaped myelin in 200 µm-long sciatic nerve fibers under a differential interference contrast (DIC) filtered microscope. Scale bar, 50 µm. (**E**) In vivo PND model using [Tg(mbp:eGFP)] zebrafish are visualized. (**F**) MBP: eGFP fluorescence in the caudal fin (green) with **3f** treatment of 1 µM for 10 days was observed under a stereomicroscope. Scale bar, 0.25 mm. (**G**) The number of intact GFP fluorescence was counted in three areas (1 × 1 mm^2^) of the distal part of the injury. * *p* < 0.05, ** *p* < 0.01, and *** *p* < 0.001.

Considering the results in the molecular docking, we subjected **3a**, **3b**, **3c**, **3e**, **3f**, and **3g** to further evaluation. To determine their ability to inhibit PND, we cultured ex vivo sciatic nerve explants for 3 days in vitro (3DIV) [20]. First, we grossly evaluated the degree of PND. Bands of Fontana are transverse stripes on surfaces around normal peripheral nerves and are absent from degenerating nerves [20]. Ex vivo, at 3DIV, only nerves treated with **3a** and **3f** showed stripes (Figure 4A,B). Ovoid-like structures in peripheral nerve fibers indicate the degree of PND because they represent myelin fragmentation [20]. At 3DIV, ovoid-like structures were not present in nerves treated with **3a** and **3f** (Figure 4C,D). These two indices also represent permeability across the blood-nerve barrier (BNB) [35]. Furthermore, **3f** was among the top three most efficacious and stable compounds in in vitro and molecular docking assays (Figure 2 and Figure 3). Although **3a** exerted an anti-neurodegenerative effect during PND, we excluded **3a** from further evaluation because of its low binding affinity for TRPA1, indicating that it targets a factor other than TRPA1.

Next, to evaluate drug efficacy in vivo, we performed morphometric analysis using embryonic and adult transgenic [Tg(mbp:eGFP)] zebrafish expressing EGFP in Schwann cells (Figure 4E) [36]. After localized injury to the peripheral nerves of the tail fin, we observed GFP fluorescence in nerves with or without **3f** treatment (Figure 4F). The control (DMSO-treated) groups did not show MBP:eGFP fluorescence in the fin, whereas the **3f**-treated groups retained fluorescence (Figure 4G). Therefore, **3f** significantly inhibited PND ex vivo and in vivo and may have greater permeability through connective layers compared with **3c** and **3e,** which were among the top three most efficacious and stable compounds in in vitro and molecular docking assays. Therefore, we further evaluated **3f** as a potential lead compound.

### 3.5. Inhibitory Phenotypes of PND Ex Vivo via ***3f***

**Figure 5:**
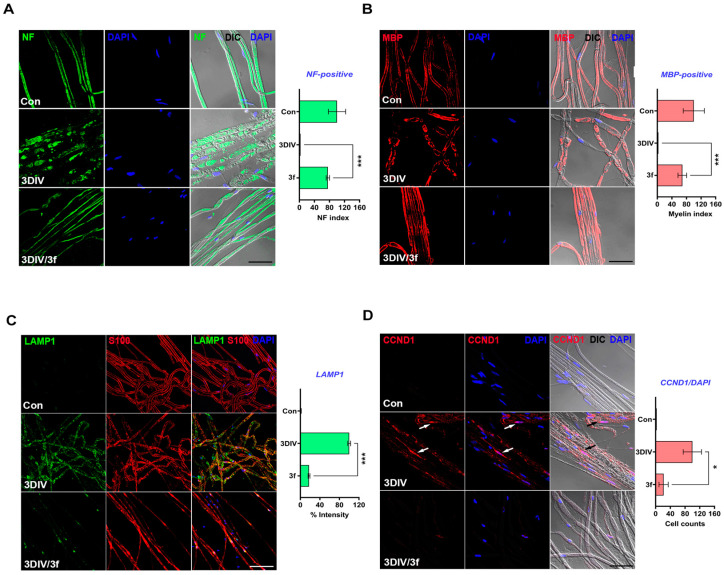
Inhibitory effect of **3f** on the phenotypes of PND. (**A**) Immunostaining was performed in explant sciatic nerve fibers. Neurofilament (NF, green) and 4′,6-Diamidine-2′-phenylindole (DAPI, blue) were used as markers for axons and nucleus, respectively. NF index was calculated by counting the 100 µm-long consecutive lines (n = 3 mice). Scale bar, 50 µm. 3DIV, 3 days in vitro. Con, control. (**B**) Myelin sheath was identified using an anti-myelin basic protein (MBP, red) antibody counterstained with DAPI. Scale bar, 50 µm. MBP index was estimated by calculating the 100 µm-long consecutive lines of MBP-marked nerve fibers (n = 3 mice). (**C**) Immunostaining with lysosomal-associated membrane protein 1 (LAMP1, green) combined with DAPI (blue) in ex vivo sciatic nerve fibers showed the inhibitory of the **3f** on Schwann cell dedifferentiation. Scale bar, 50 µm. The intensity of LAMP1 expression was quantitatively measured in the teased sciatic nerve fibers within 200 × 200 μm^2^ widths of teasing slides (n = 3 mice). The intensity at 3DIV was set as 100%. (**D**) Cyclin D1 (CCND1, red) and DAPI (blue) were immunostained in ex vivo sciatic nerve fibers. The arrows indicated CCND1/DAPI double-positive cells. Scale bar, 50 µm. Quantification was proceeded by calculating CCND1-marked cells out of 200 DAPI-positive cells and then compared to that of the nerve fibers at 3DIV (n = 3 mice). * *p* < 0.05, *** *p* < 0.001.

To determine whether Schwann cells are targeted by **3f**, we performed an immunochemical analysis of the biomarkers of PND. Degenerating nerve fibers show axon degradation and Schwann cell demyelination, proliferation, and dedifferentiation [37,38]. These are regulated in or by Schwann cells during PND. NF is an axon marker [39], and we performed immunostaining for NF in sciatic nerve samples to assess the inhibitory effect of **3f** on axonal degradation. Moreover, **3f** maintained NF structures in nerve fibers (Figure 5A). MBP is a marker of myelination/demyelination in Schwann cells [39]. At 3DIV, **3f** is protected against myelin sheath breakdown by Schwann cells during PND (Figure 5B). LAMP1 is a lysosomal membrane protein and a marker of Schwann cell dedifferentiation [39]. At 3DIV, **3f**-treated nerve fibers showed suppressed LAMP1 expression in Schwann cells compared with untreated nerve fibers (Figure 5C). Lastly, we performed immunostaining using a CCND1-specific antibody as a marker of cell proliferation [39]. Here, **3f** inhibited Schwann cell proliferation (Figure 5D). Therefore, **3f** inhibited the Schwann cell phenotypes and could be a lead compound having therapeutic potential for PND in vitro, ex vivo, and in vivo.

### 3.6. Pharmacological Mechanism of ***3f***

To assess the mechanism by which **3f** inhibits PND, we bioinformatically investigated the expression of coding genes and miRNAs regulated by CAH by whole-transcriptome sequencing using SK-MES-1 and NCI-H226 non-small cell lung cancer cell lines [21], which exhibit proliferation, dedifferentiation, and an epithelial-mesenchymal transition (cell movement) similar to those properties of Schwann cells [40].

**Figure 6:**
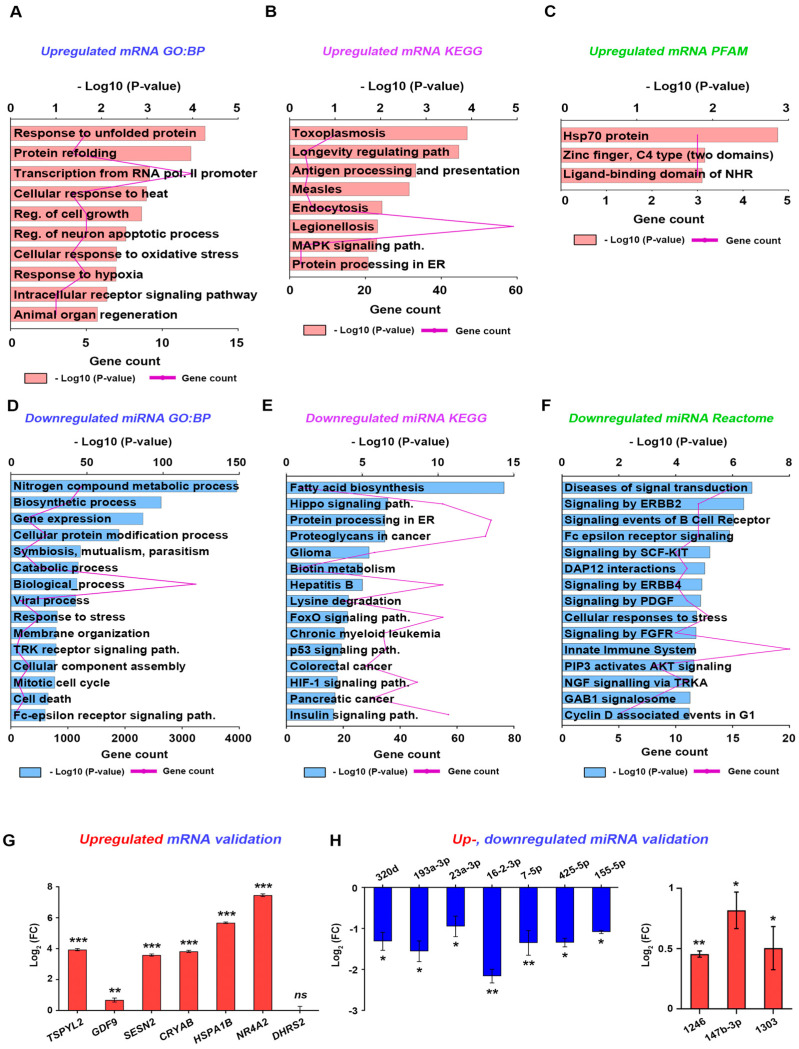
In silico analysis for the pharmacological mechanism of **3f** and the validation in hTERT02.3 cells. Bioinformatics analysis using 150 DEGs or 21 DE miRNAs regulated by CAH in non-small cell lung cancer cell lines (NSCLC: SK-MES-1 and NCI-H226 cell lines) [11] was performed. (**A**,**D**) Gene ontology (GO), (**B**,**E**) Kyoto encyclopedia of genes and genomes (KEGG), and (**C**,**F**) protein domain analyses were performed using DAVID bioinformatics resources (https://david.ncifcrf.gov/; accessed on 10 March 2022) and PFAM protein family database (https://pfam.xfam.org/; accessed on 10 March 2022). The bar graph represented the value of ‘−log (*p*-value)’, and the line graph showed the gene counts. (**G**) The mRNA levels of seven coding genes related to cell growth and oxidative stress were validated in hTERT02.3 cells with or without **3f** treatment by qPCR. ** *p* < 0.01 and *** *p* < 0.001. (**H**) The miRNA levels of 10 miRNAs related to cell cycle were validated in hTERT02.3 cells by qPCR. * *p* < 0.05 and ** *p* < 0.01.

First, 150 differentially expressed genes (DEGs), comprising 81 upregulated and 69 downregulated genes, were obtained [21]. To identify their functional enrichments, we performed GO term, KEGG pathway, and protein domain enrichment analyses. Among the top 10 GO terms in the biological process were negative regulation of megakaryocyte differentiation, animal organ regeneration, regulation of cell growth, regulation of neuron apoptotic process, and cellular response to oxidative stress (Figure 6A and Figure S3A). Among the top KEGG pathways, the MAPK signaling pathway, transcriptional dysregulation in cancer, endocytosis, and antigen processing and presentation were significantly enriched (Figure 6B and Figure S3B). The protein domains included the zinc finger/C4 type (two domains), the ligand-binding domain of NHR, and the kinetochore component CENP-T (Figure 6C and Figure S3C). The top GO terms in the cellular component and molecular function using DEGs were enriched in Figure S4. Therefore, these results indicated that CAH regulates cell proliferation, growth, differentiation, response to oxidative stress, and transcriptional regulation, which are linked to Schwann cell phenotypes during PND [36,41].

Next, 21 DE miRNAs, comprising 11 upregulated and 10 downregulated miRNAs, were obtained [21]. GO term analysis classified the 21 miRNAs into the top 15 GO functional terms according to their P-values. The genes targeted by the miRNAs were significantly abundant in the biological processes; cellular nitrogen compound metabolic process, catabolic process, response to stress, and mitotic cell cycle (Figure 6D and Figure S5A). Most of the top 15 representative KEGG pathways were related to Schwann cell proliferation, myelination and differentiation, fatty acid biosynthesis, and the FoxO, insulin, mTOR, and Hippo pathways [42,43,44] (Figure 6E and Figure S5B). The top 15 Reactome also highlighted Schwann cell proliferation, differentiation, and myelination, signaling by ERBB2, signaling by PDGF, PIP3 activating AKT signaling, and cyclin D-associated events in G1 [45,46,47] (Figure 6F and Figure S5C). Cell components and molecular function using DE miRNAs in GO terms were also enriched (Figure S6). Collectively, CAH affects cell proliferation, cell differentiation, and the response to oxidative stress at transcriptional or post-transcriptional levels.

We performed qPCR to determine how the DEGs and DE miRNAs regulated by CAH are expressed in Schwann cells exposed to **3f**. Among the 150 DEGs, the expression of 7 associated with cellular response to oxidative stress and negative regulation of cell growth were evaluated in hTERT02.3, human Schwann cells. TSPYL2, GDF9, SESN2, CRYAB, HSPA1B, and NR4A2 were upregulated, and DHRS2 was unchanged in **3f**-treated hTERT02.3 cells compared to the control (Figure 6G). Among the 21 miRNAs, the expression patterns of 10 associated with the mitotic cell cycle were evaluated in hTERT02.3 with or without **3f** treatment. hsa-miR-1246, hsa-miR-147b-3p, and hsa-miR-1303 were upregulated; hsa-320d, hsa-miR-193-3p, hsa-miR-23a-3p, hsa-miR-16-2-3p, hsa-miR-7-5p, hsa-miR-425-5p, and hsa-miR-155-5p were downregulated in **3f**-treated hTERT02.3 cells (Figure 6H).

### 3.7. In Vivo Embryo and Adult Zebrafish Toxicity

**Figure 7:**
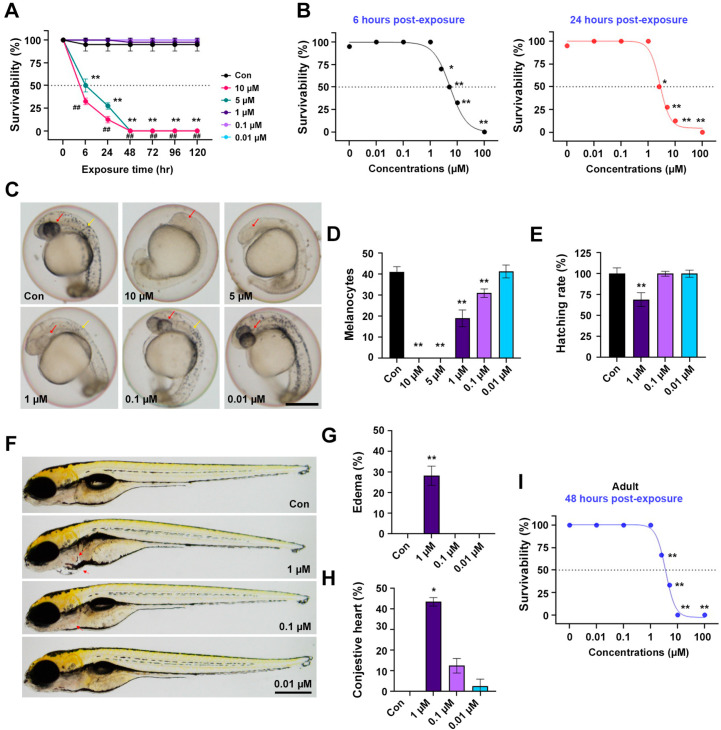
In vivo toxicity assay of **3f** in the embryo and adult zebrafish. (**A**) Time-dependent survival rates throughout 0–120 h post exposure (hpe) in zebrafish embryos (n = 25 embryos/group). * *p* < 0.05, ** *p* < 0.01 compared to control and ## *p* < 0.01 compared to 5 hpe groups. (**B**) Concentration-dependent survival curves of **3f** after continuous exposure for 6 h (left panel, black) and 24 h (right panel, red) in zebrafish embryos (n = 25 embryos/group). * *p* < 0.05 and ** *p* < 0.01. (**C**) Representative images of melanocyte development in zebrafish embryos exposed to **3f** at 24 hpe. Scale bar, 0.25 mm. (**D**) The number of melanocytes in the head and trunk of zebrafish embryos was counted in three experiments. ** *p* < 0.01. (**E**) Hatching rate of zebrafish embryos after 48 h exposure to **3f** (n = 25 embryos/group) was calculated. ** *p* < 0.01. (**F**) Malformation of zebrafish larvae exposed to **3f** at 120 hpe was estimated. **3f**-exposed zebrafish (1 µM) showed pericardial edema and congestive heart (red arrow). Scale bar, 0.5 mm. Quantification of pericardial edema (**G**) and congestive heart (**H**) in zebrafish larvae exposed to **3f** for 120 h (n = 25 embryos/group) were shown. * *p* < 0.05 and ** *p* < 0.01. (**I**) Concentration-dependent survival curves of **3f** after 3 consecutive days of exposure in adult zebrafish (n = 25 embryos/group) were displayed. Error bars indicated the standard deviation of the mean. ** *p* < 0.01.

The acute systemic toxicity of **3f** was assessed in zebrafish. Zebrafish embryos were exposed to a high dose of **3f** at 4 h post-fertilization (hpf), and survival was monitored every 24 h. Overall, **3f** at >1 µM resulted in severe embryotoxicity at 6 h post-exposure (hpe), and all embryos died at 24 hpe (Figure 7A). The 50% lethal concentration (LC_50_) values of **3f** were 5.36 and 2.69 µM at 6 and 24 hpe, respectively (Figure 7B). At 24 hpe, **3f** at >5 µM caused early embryonic death with severe developmental delays (Figure 7C). Zebrafish melanocytes are derived from neural crest cells, which are highly migratory. Because Schwann cells originate from neural crest cells [48], we can assess Schwann cell development by monitoring melanocyte migration in embryos. Exposure to **3f** prevented melanocyte development and migration in a concentration-dependent manner (Figure 7D). The hatching rate was slightly decreased by 1 µM **3f** at 48 hpe (Figure 7E). In addition, long-term exposure (120 hpe) to **3f** at 1 µM induced morphological abnormalities such as edema and congestive heart failure (Figure 7F,G). The cytotoxic effect of **3f** at 48 hpe was confirmed in adult (5-month-old) zebrafish by the LD_50_ value of 3.58 µM (Figure 7I). Therefore, **3f** has cardiac toxicity in zebrafish embryos and cytotoxicity in adult zebrafish. However, the toxicity of **3f** could be resolved in the lead optimization phase via clever changing functional groups by medicinal chemists.

## 4. Conclusions

Systemic peripheral nerve injuries such as hyperglycemic conditions induce irreversible PND, and as a result, nerve regeneration does not generate (irreversible nerve degeneration). In non-curable cases such as diabetic peripheral neuropathy, an effective therapeutic strategy is to delay or stop nerve degeneration. Thus, we focused on determining effective synthetic drugs to delay or stop PND, and we think that our CAH derivatives could be viable candidates. Here, we designed and synthesized compounds targeting Schwann cells for PND. Nothing has been known about synthetic compounds for PND or medicines for peripheral neurodegenerative diseases via systematic drug design and pharmaceutical investigation despite the high prevalence rate of peripheral neurodegenerative diseases. Most newly synthesized CAH derivatives significantly inhibited in vitro Schwann cell proliferation. Among them, **3f** had the highest in vitro, ex vivo, and in vivo drug efficacy and could cross the BNB in peripheral nerves. A molecular docking study showed that several compounds with large aromatic rings (e.g., naphthalene) had higher binding affinities for TRPA1 than CAH, and their interaction was dependent on hydrophobic interactions. Coding genes and miRNAs regulated by CAH were enriched for cell proliferation, differentiation, and response to oxidative stress and based on the results, the pharmacological activity of **3f** was inferred.

Conversely, our CAH derivatives are α,β-unsaturated carbonyl compounds and could act as irreversible/reversible covalent Michael acceptors [49]. However, these compounds could also bind with TRPA1 by hydrophobic interaction because they contain aromatic rings. The steric effects of their phenyl or naphthyl groups structurally inhibit the Michael reaction between the derivatives and cysteine residues of target proteins. Hydrolysis of benzenesulfonate occurs and leads to sulfonates and phenolates [50]. This property may reduce the pharmacokinetic ability of **3f** in the human body. However, our disease model utilizes ex vivo sciatic nerves which are wrapped with three-layered connective tissues and thus, to penetrate the barriers [BNB], the drug requires strong hydrophobicity. Among the 12 derivatives, CAH and CL, **3f** is highly hydrophobic and contains more bulky substitutes than others. Thus, **3f** may become the best lead compound among the 12 derivatives in the peripheral nervous system. We expanded the chemical library of CAH scaffolds and validated their utility in vitro, ex vivo, in vivo, and in silico. The results will facilitate the development of novel synthetic multi-target drugs for peripheral neurodegenerative diseases targeting TRPA1 in Schwann cells.

## Data Availability

Data is contained within the article or supplementary materials.

## Supplementary Materials

The following supporting information can be downloaded at: https://www.mdpi.com/article/10.3390/antiox11101846/s1, Supplementary synthetic procedure; NMR Spectra; Figure S1: Therapeutic indices of CAH derivatives in dedifferentiated SW10 cells; Figure S2: Binding affinity between protein targets of chalcone (CL) and **3f**; Figure S3: Gene ontology (GO), Kyoto Encyclopedia of Genes and Genomes (KEGG), and Protein Family (PFAM) assays using downregulated differentially expressed genes (DEGs); Figure S4: GO molecular function (MF) and cellular component (CC) analysis using upregulated and downregulated DEGs; Figure S5: GO terms, KEGG pathway, and Reactome assays using upregulated differentially expressed miRNAs (DE miRNAs); Figure S6: GO molecular function (MF) and cellular component (CC) analysis using upregulated and downregulated DE miRNAs; Table S1: Primer sequences used in qPCR validation; Table S2: Molecular docking between TRPA1 and CAH derivatives.
