# Supplementary material for: Novel Cinnamaldehyde Derivatives Inhibit Peripheral Nerve Degeneration by Targeting Schwann Cells
Source: Antioxidants (Basel). 2022 Sep 20;11(10):1846. doi: 10.3390/antiox11101846 (PMC9598575; doi:10.3390/antiox11101846)

Supplementary Material 1

Supplementary synthetic procedure

1. General information

NMR spectra were recorded on Bruker BioSpin spectrometers operating at 300.13 MHz for ^{1}H NMR and for ^{13}C NMR, Bruker BioSpin spectrometers, operating at 126 MHz and 75MHz. Chemical shifts were reported in the scale relative to TMS (0.00 ppm for ^{1}H NMR), $CDCl_3$ (7.26 ppm for ^{1}H NMR), and $CDCl_3$ (77.16 ppm for ^{13}C NMR) as an internal reference, respectively. Silica gel column chromatography was performed with Kanto Silica gel 60 N (40-50 mesh). TLC analysis was carried out on Merck Kieselgel 60 F_{254} plates with visualization by UV light.

2. General procedure to synthesize CAH derivatives

2.1. (*E*)-3-(2-(allyloxy)phenyl)-1-phenylprop-2-en-1-one (**3a**)

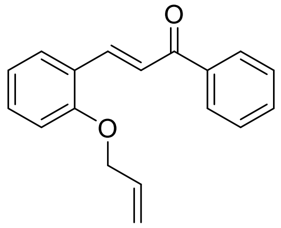

Round-bottomed flask was charged with acetophenone (0.24gm, 2.0 mmol) and 2-(allyloxy) benzaldehyde (0.32gm, 2.0 mmol), and mixed with 5 mL methanol. NaOH (0.16gm, 4.0 mmol) was dissolved in 5mL of a methanol: water mixture (1:1) and then added to the methanolic solution of ketone and aldehyde. Then reaction mixture was allowed to stir at room temperature. The crude residue was purified by column chromatography using 15% hexane: ethyl acetate as eluents to afford desired product **3a** as a white solid.

^{1}H NMR (300 MHz, $CDCl_3$) δ 8.18 (d, J = 15.9 Hz, 1H), 8.09 – 7.97 (m, 2H), 7.74 – 7.63 (m, 2H), 7.63 – 7.56 (m, 1H), 7.52 (ddt, J = 8.5, 6.6, 1.4 Hz, 2H), 7.37 (ddd, J = 8.3, 7.4, 1.7 Hz, 1H), 7.02 (td, J = 7.5, 1.1 Hz, 1H), 6.95 (dd, J = 8.3, 1.1 Hz, 1H), 6.13 (ddt, J = 17.1, 10.5, 5.2 Hz, 1H), 5.48 (dq, J = 17.3, 1.6 Hz, 1H), 5.35 (dq, J = 10.5, 1.4 Hz, 1H), 4.66 (dt, J = 5.2, 1.5 Hz, 2H). ^{13}C NMR (75 MHz, $CDCl_3$) δ 191.1, 157.8, 140.5, 138.5, 132.8, 132.6, 131.7, 129.4, 128.5, 128.5, 124.2, 123.0, 121.0, 117.9, 112.5, 69.2.

2.2. (*E*)-2-(3-(2-(allyloxy)phenyl)acryloyl)cyclohexan-1-one (**3b**)

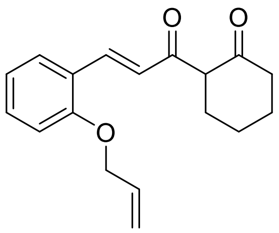

Round-bottomed flask was charged with 2-acetylcyclohexanone (0.28gm, 2.0 mmol) and 2-(allyloxy)benzaldehyde (0.32gm, 2.0 mmol), and mixed with 5 mL methanol. NaOH (0.16gm, 4.0 mmol) was dissolved in 5mL of a methanol: water mixture (1:1) and then added to the methanolic solution of ketone and aldehyde. Then reaction mixture was allowed to stir at room temperature. The crude residue was purified by column chromatography using 13% hexane: ethyl acetate as eluents to afford desired product **3b** as a pale white solid.

^{1}H NMR (300 MHz, $CDCl_3$) δ 8.06 (d, *J* = 2.1 Hz, 1H), 7.45 – 7.18 (m, 3H), 7.08 – 6.79 (m, 2H), 6.25 – 5.93 (m, 1H), 5.50 – 5.37 (m, 1H), 5.29 (ddd, *J* = 10.5, 3.6, 1.7 Hz, 1H), 4.60 (dd, *J* = 6.4, 4.8 Hz, 2H), 2.82 (m, 2H), 2.56 (t, *J* = 6.7 Hz, 1H), 2.10 – 1.89 (m, 2H), 1.75 (ddd, *J* = 12.0, 6.7, 2.0 Hz, 2H), 1.28 (t, *J* = 7.1 Hz, 1H). ^{13}C NMR (75 MHz, $CDCl_3$) δ 202.0, 190.3, 157.3, 136.9, 136.4, 133.1, 133.1, 132.5, 131.1, 130.4, 130.4, 129.9, 129.8, 125.5, 124.9, 120.09, 120.07, 117.5, 112.09, 112.02, 69.1, 60.4, 40.5, 28.8, 24.0, 23.4.

2.3. (*E*)-1-(naphthalen-1-yl)-3-(2-nitrophenyl)prop-2-en-1-one (**3c**)

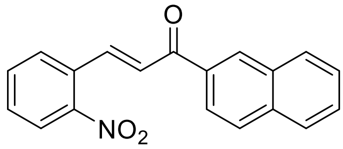

Round-bottomed flask was charged with 1-acetonaphthone (0.34gm, 2.0 mmol) and 2-nitrobenzaldehyde (0.30gm, 2.0 mmol), and mixed with 5 mL methanol. NaOH (0.16gm, 4.0 mmol) was dissolved in 5mL of a methanol: water mixture (1:1) and then added to the methanolic solution of ketone and aldehyde. Then reaction mixture was allowed to stir at room temperature. The crude residue was purified by column chromatography using 20% hexane: ethyl acetate as eluents to afford desired product **3c** as a pale yellow solid.

^{1}H NMR (300 MHz, $CDCl_3$) δ 8.45 – 8.37 (m, 1H), 8.13 – 8.02 (m, 3H), 8.00 – 7.94 (m, 1H), 7.94 – 7.88 (m, 2H), 7.79 – 7.67 (m, 2H), 7.59 (ddt, *J* = 8.5, 7.0, 2.8 Hz, 4H), 7.17 (d, *J* = 15.9

Hz, 1H). ^{13}C NMR (75 MHz, $CDCl_3$) δ 195.1, 148.4, 141.4, 135.7, 133.9, 133.6, 132.2, 131.7, 131.1, 130.5, 130.4, 129.2, 128.5, 128.1, 127.7, 126.6, 125.5, 125.0, 124.4.

2.4. (*E*)-3-(2-(allyloxy)phenyl)-1-(4-aminophenyl)prop-2-en-1-one (**3d**)

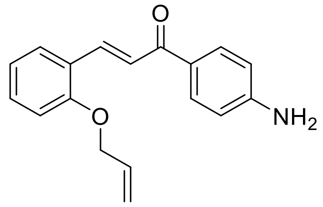

Round-bottomed flask was charged with 4-aminoacetophenone (0.27gm, 2.0 mmol) and 2-(allyloxy)benzaldehyde (0.32gm, 2.0 mmol), and mixed with 5 mL methanol. NaOH (0.16gm 4.0 mmol) was dissolved in 5mL of a methanol: water mixture (1:1) and then a, ded to the methanolic solution of ketone and aldehyde. Then reaction mixture was allowed to stir at room temperature. The crude residue was purified by column chromatography using 15% hexane: ethyl acetate as eluents to afford desired product **3d** as a red solid.

^{1}H NMR (300 MHz, $CDCl_3$) δ 8.13 (d, *J* = 15.8 Hz, 1H), 8.00 – 7.91 (m, 2H), 7.85 – 7.80 (m, 1H), 7.74 – 7.61 (m, 2H), 7.35 (ddd, *J* = 8.5, 7.4, 1.7 Hz, 1H), 7.05 – 6.91 (m, 2H), 6.80 – 6.71 (m, 2H), 6.22 – 6.00 (m, 1H), 5.48 (dq, *J* = 17.3, 1.6 Hz, 1H), 5.40 – 5.27 (m, 1H), 4.65 (dt, *J* = 5.3, 1.6 Hz, 2H), 4.12 (s, 2H). ^{13}C NMR (75 MHz, $CDCl_3$) δ 188.7, 157.6, 151.2, 138.6, 132.9, 131.1, 130.8, 129.2, 128.5, 124.5, 123.0, 120.9, 117.9, 114.0, 112.5, 69.2.

2.5. (*E*)-3-(2-(allyloxy)phenyl)-1-(naphthalen-2-yl)prop-2-en-1-one (**3e**)

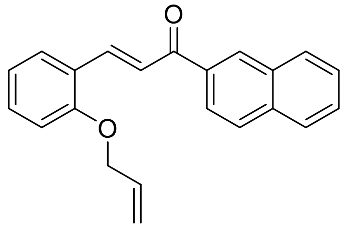

Round-bottomed flask was charged with 2-acetonaphthanone (0.34gm, 2.0 mmol) and 2-(allyloxy)benzaldehyde (0.32gm, 2.0 mmol), and mixed with 5 mL methanol. NaOH (0.16gm, 4.0 mmol) was dissolved in 5mL of a methanol: water mixture (1:1) and then added to the methanolic solution of ketone and aldehyde. Then reaction mixture was allowed to stir at room temperature. The crude residue was purified by column chromatography using 18% hexane:

ethyl acetate as eluents to afford desired product **3e** as an off-white solid.
^{1}H NMR (300 MHz, $CDCl_3$) δ 8.42 – 8.30 (m, 1H), 8.06 – 8.01 (m, 1H), 7.99 (d, *J* = 3.2 Hz, 1H), 7.96 – 7.90 (m, 1H), 7.80 (dd, *J* = 7.1, 1.2 Hz, 1H), 7.63 (dd, *J* = 7.7, 1.7 Hz, 1H), 7.60 – 7.51 (m, 3H), 7.41 – 7.33 (m, 2H), 7.01 (td, *J* = 7.6, 1.1 Hz, 1H), 6.93 (dd, *J* = 8.4, 1.1 Hz, 1H), 6.01 (ddt, *J* = 17.2, 10.3, 5.0 Hz, 1H), 5.40 – 5.28 (m, 1H), 5.25 (dt, *J* = 10.6, 1.4 Hz, 1H), 4.60 (dt, *J* = 5.1, 1.6 Hz, 2H). ^{13}C NMR (75 MHz, $CDCl_3$) δ 196.4, 157.6, 141.6, 137.3, 133.8, 132.6, 131.9, 131.3, 130.6, 129.0, 128.4, 127.7, 127.3, 127.1, 126.3, 125.8, 124.5, 123.9, 121.03, 117.6, 112.6, 69.0.

2.6. (*E*)-2-(3-(naphthalen-2-yl)-3-oxoprop-1-en-1-yl)phenyl benzenesulfonate (**3f**)

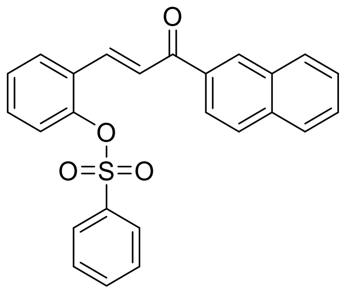

Round-bottomed flask was charged with 2-acetonaphthanone (0.34gm, 2.0 mmol) and 2-formylphenyl benzenesulfonate (0.52gm, 2.0 mmol), and mixed with 5 mL methanol. NaOH (0.16gm, 4.0 mmol) was dissolved in 5mL of a methanol: water mixture (1:1) and then added to the methanolic solution of ketone and aldehyde. Then reaction mixture was allowed to stir at room temperature. The crude residue was purified by column chromatography using 20% hexane: ethyl acetate as eluents to afford desired product **3f** as a white solid.
^{1}H NMR (300 MHz, $CDCl_3$) δ 8.39 – 8.29 (m, 1H), 8.02 (d, *J* = 8.2 Hz, 2H), 7.97 – 7.91 (m, 2H), δ 7.89 (s, 1H), 7.84 – 7.66 (m, 3H), 7.63 – 7.50 (m, 4H), 7.45 (dd, *J* = 7.8, 1.6 Hz, 1H), 7.31 – 7.21 (m, 2H), 7.00 (dd, *J* = 8.2, 1.1 Hz, 1H), 6.91 (td, *J* = 7.5, 1.1 Hz, 1H). ^{13}C NMR (75 MHz, $CDCl_3$) δ 198.5, 156.3, 143.7, 136.9, 133.8, 132.1, 131.5, 130.6, 130.4, 129.8, 128.4, 127.4, 127.3, 126.4, 125.7, 124.5, 121.7, 120.7, 119.7, 117.9, 117.4, 116.8.

2.7. (*E*)-3-(2-(allyloxy)-5-methoxyphenyl)-1-(naphthalen-2-yl)prop-2-en-1-one (**3g**)

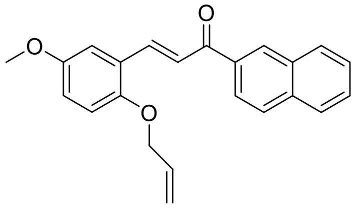

Round-bottomed flask was charged with 2-acetonaphthone (0.34gm, 2.0 mmol) and 2-(allyloxy)-5-methoxybenzaldehyde (0.38gm, 2.0 mmol), and mixed with 5 mL methanol. NaOH (0.16gm, 4.0 mmol) was dissolved in 5mL of a methanol: water mixture (1:1) and then added to the methanolic solution of ketone and aldehyde. Then reaction mixture was allowed to stir at room temperature. The crude residue was purified by column chromatography using 18% hexane: ethyl acetate as eluents to afford desired products **3g** as a colorless solid.

^{1}H NMR (300 MHz, $CDCl_3$) δ 8.41 – 8.31 (m, 1H), 8.06 – 7.90 (m, 3H), 7.80 (dd, J = 7.1, 1.3 Hz, 1H), 7.64 – 7.52 (m, 3H), 7.38 (d, J = 16.2 Hz, 1H), 7.16 (d, J = 2.9 Hz, 1H), 6.94 (dd, J = 9.0, 3.0 Hz, 1H), 6.87 (d, J = 9.0 Hz, 1H), 5.98 (ddt, J = 17.3, 10.4, 5.1 Hz, 1H), 5.33 – 5.25 (m, 1H), 5.24 (s, 1H), 4.53 (dt, J = 5.1, 1.6 Hz, 2H), 3.82 (s, 3H). ^{13}C NMR (75 MHz, $CDCl_3$) δ 196.3, 153.7, 152.1, 141.4, 137.2, 133.8, 132.9, 131.4, 130.6, 128.4, 127.9, 127.3, 127.1, 126.4, 125.8, 124.6, 124.5, 117.7, 117.4, 114.3, 113.0, 69.9, 55.8.

2.8. (*E*)-1-(4-aminophenyl)-3-(2-((3-methylbut-2-en-1-yl)oxy)phenyl)prop-2-en-1-one (**3h**)

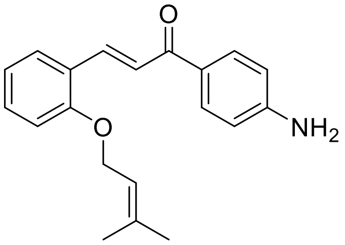

Round-bottomed flask was charged with 4-aminoacetophenone (0.27gm, 2.0 mmol) and 2-((3-methylbut-2-en-1-yl)oxy)benzaldehyde (0.38gm, 2.0 mmol), and mixed with 5 mL methanol. NaOH (0.16gm, 4.0 mmol) was dissolved in 5mL of a methanol: water mixture (1:1) and then added to the methanolic solution of ketone and aldehyde. Then reaction mixture was allowed to stir at room temperature. The crude residue was purified by column chromatography using 16% hexane: ethyl acetate as eluents to afford desired product **3h** as a red solid.

^{1}H NMR (300 MHz, $CDCl_3$) δ 8.06 (d, J = 15.7 Hz, 1H), 7.98 – 7.92 (m, 1H), 7.84 (d, J = 2.0 Hz, 2H), 7.82 (d, J = 2.0 Hz, 3H), 7.62 (dd, J = 7.6, 1.7 Hz, 1H), 7.35 (ddd, J = 8.2, 7.4, 1.8 Hz, 1H), 7.04 – 6.93 (m, 2H), 6.73 (d, J = 1.8 Hz, 1H), 6.68 (d, J = 2.0 Hz, 2H), 6.66 (d, J = 1.9 Hz, 2H), 5.65 – 5.53 (m, 1H), 4.63 (d, J = 6.8 Hz, 2H), 1.85 (d, J = 1.4 Hz, 3H), 1.80 – 1.75 (m, 3H). ^{13}C NMR (126 MHz, $CDCl_3$) δ 188.9, 163.3, 158.3, 143.7, 140.5, 133.2, 131.6, 130.8, 129.3, 126.8, 123.7, 122.8, 119.7, 117.2, 113.8, 113.4, 55.5, 35.1, 29.7.

2.9. (*E*)-3-(2-(allyloxy)-3-(tert-butyl)phenyl)-1-(4-methoxyphenyl)prop-2-en-1-one (**3i**)

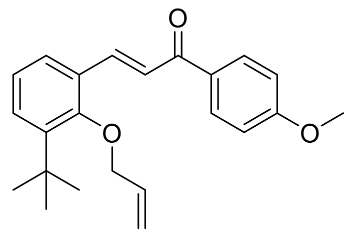

Round-bottomed flask was charged with 4-methoxyacetophenone (0.30gm, 2.0 mmol) and 2-(allyloxy)-3-(tert-butyl)benzaldehyde (0.43gm 2.0 mmol), and mixed with 5 mL methanol. NaOH (0.16gm, 4.0 mmol) was dissolved in 5mL of a methanol: water mixture (1:1) and then added to the methanolic solution of ketone and aldehyde. Then reaction mixture was allowed to stir at room temperature. The crude residue was purified by column chromatography using 15% hexane: ethyl acetate as eluents to afford desired product **3i** as a colorless gum.

^{1}H NMR (300 MHz, $CDCl_3$) δ 8.08 (d, *J* = 2.0 Hz, 1H), 8.04 (d, *J* = 4.9 Hz, 1H), 7.82 – 7.72 (m, 1H), 7.63 – 7.53 (m, 2H), 7.43 (dd, *J* = 7.9, 1.7 Hz, 1H), 7.16 – 7.07 (m, 1H), 7.02 (d, *J* = 2.1 Hz, 1H), 6.99 (t, *J* = 2.4 Hz, 1H), 6.24 – 6.04 (m, 1H), 5.66 – 5.48 (m, 1H), 5.42 – 5.26 (m, 1H), 4.42 (dt, *J* = 5.0, 1.7 Hz, 2H), 3.92 (d, *J* = 3.6 Hz, 3H), 1.45 (d, *J* = 1.9 Hz, 9H). ^{13}C NMR (126 MHz, $CDCl_3$) δ 189.3, 163.3, 158.3, 143.7, 140.5, 133.2, 131.6, 130.8, 129.3, 126.8, 123.7, 122.8, 117.2, 113.8, 113.4, 75.9, 55.5, 35.1, 29.7.

2.10. (*E*)-3-(2-(allyloxy)-3-(tert-butyl)phenyl)-1-phenylprop-2-en-1-one (**3j**)

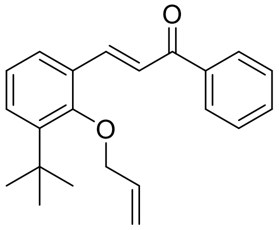

Round-bottomed flask was charged with acetophenone (0.24gm, 2.0 mmol) and and 2-(allyloxy)-3-(tert-butyl)benzaldehyde (0.43gm 2.0 mmol), and mixed with 5 mL methanol. NaOH (0.16gm, 4.0 mmol) was dissolved in 5mL of a methanol: water mixture (1:1) and then added to the methanolic solution of ketone and aldehyde. Then reaction mixture was allowed to stir at room temperature. The crude residue was purified by column chromatography using 16% hexane: ethyl acetate as eluents to afford desired products **3j** as a white solid.

^{1}H NMR (300 MHz, $CDCl_3$) δ 8.05 – 7.98 (m, 3H), 7.62 – 7.54 (m, 2H), 7.49 (d, *J* = 6.4 Hz, 2H), 7.44 (d, *J* = 1.3 Hz, 1H), 7.29 – 7.22 (m, 1H), 7.16 (dd, *J* = 7.7, 1.7 Hz, 1H), 7.02 (t, *J* = 7.7 Hz, 1H), 6.03 (ddt, *J* = 17.2, 10.5, 4.8 Hz, 1H), 5.39 (dq, *J* = 17.2, 1.8 Hz, 1H), 5.23 (dq, *J* = 10.5, 1.6 Hz, 1H), 4.53 – 4.43 (m, 3H), 3.40 (dd, *J* = 8.5, 7.1 Hz, 4H), 1.41 (s, 9H). ^{13}C NMR (75 MHz, $CDCl_3$) δ 198.9, 155.9, 143.6, 137.6, 136.9, 133.8, 133.0, 128.6, 128.2, 126.0, 124.7, 124.0, 116.3, 74.9, 44.4, 35.3, 31.2.

2.11. (*E*)-3-(2-(allyloxy)-3,5-dibromophenyl)-1-phenylprop-2-en-1-one (**3k**)

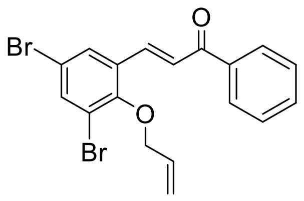

Round-bottomed flask was charged with acetophenone (0.24gm, 2.0 mmol) and 2-(allyloxy)-3,5-dibromobenzaldehyde (0.63gm, 2.0 mmol), and mixed with 5 mL methanol. NaOH (0.16gm, 4.0 mmol) was dissolved in 5mL of a methanol: water mixture (1:1) and then added to the methanolic solution of ketone and aldehyde. Then reaction mixture was allowed to stir at room temperature. The crude residue was purified by column chromatography using 18% hexane: ethyl acetate as eluents to afford desired product **3k** as a reddish brown solid.

^{1}H NMR (300 MHz, $CDCl_3$) δ 8.04 – 7.97 (m, 3H), 7.61 – 7.55 (m, 2H), 7.51 (s, 1H), 7.47 (d,

J = 7.1 Hz, 2H), 7.31 (d, J = 2.3 Hz, 1H), 6.15 (m, 1H), 5.43 (dq, J = 17.1, 1.6 Hz, 1H), 5.28 (dq, J = 10.3, 1.4 Hz, 1H), 4.63 (dt, J = 5.8, 1.4 Hz, 2H). ^{13}C NMR (126 MHz, $CDCl_3$) δ 198.0, 153.0, 141.2, 136.5, 134.9, 134.0, 133.3, 133.2, 132.8, 129.2, 128.7, 128.2, 118.9, 118.3, 117.5, 74.3.

2.12. (*E*)-3-(2-(allyloxy)-3,5-dichlorophenyl)-1-(4-aminophenyl)prop-2-en-1-one (3l)

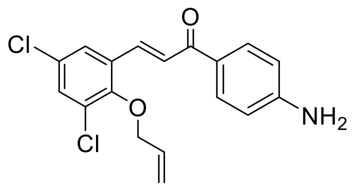

Round-bottomed flask was charged with 4-Aminoacetophenone (0.27gm, 2.0 mmol) and 2-(allyloxy)-3,5-dichlorobenzaldehyde (0.46gm, 2.0 mmol), and mixed with 5 mL methanol. NaOH (0.16gm, 4.0 mmol) was dissolved in 5mL of a methanol: water mixture (1:1) and then added to the methanolic solution of ketone and aldehyde. Then reaction mixture was allowed to stir at room temperature. The crude residue was purified by column chromatography using 18% hexane: ethyl acetate as eluents to afford desired products **3l** as a pale yellow solid.

^{1}H NMR (300 MHz, $CDCl_3$) δ 8.01 – 7.76 (m, 3H), 7.62 (d, J = 15.7 Hz, 1H), 7.55 (d, J = 2.6 Hz, 1H), 7.42 (d, J = 2.4 Hz, 1H), 6.88 – 6.57 (m, 2H), 6.24 – 5.99 (m, 1H), 5.42 (dq, J = 17.1, 4.8, 1.5 Hz, 1H), 5.30 (dq, J = 10.2, 7.5, 1.4 Hz, 1H), 4.58 – 4.43 (m, 2H), 4.30 – 3.67 (m, 2H). ^{13}C NMR (75 MHz, $CDCl_3$) δ 187.5, 152.7, 151.4, 136.0, 132.5, 132.2, 131.2, 131.0, 129.9, 129.7, 128.0, 126.4, 125.4, 119.2, 113.9, 75.2.

NMR Spectra

¹H NMR for 3a

(*E*)-3-(2-(allyloxy)phenyl)-1-phenylprop-2-en-1-one

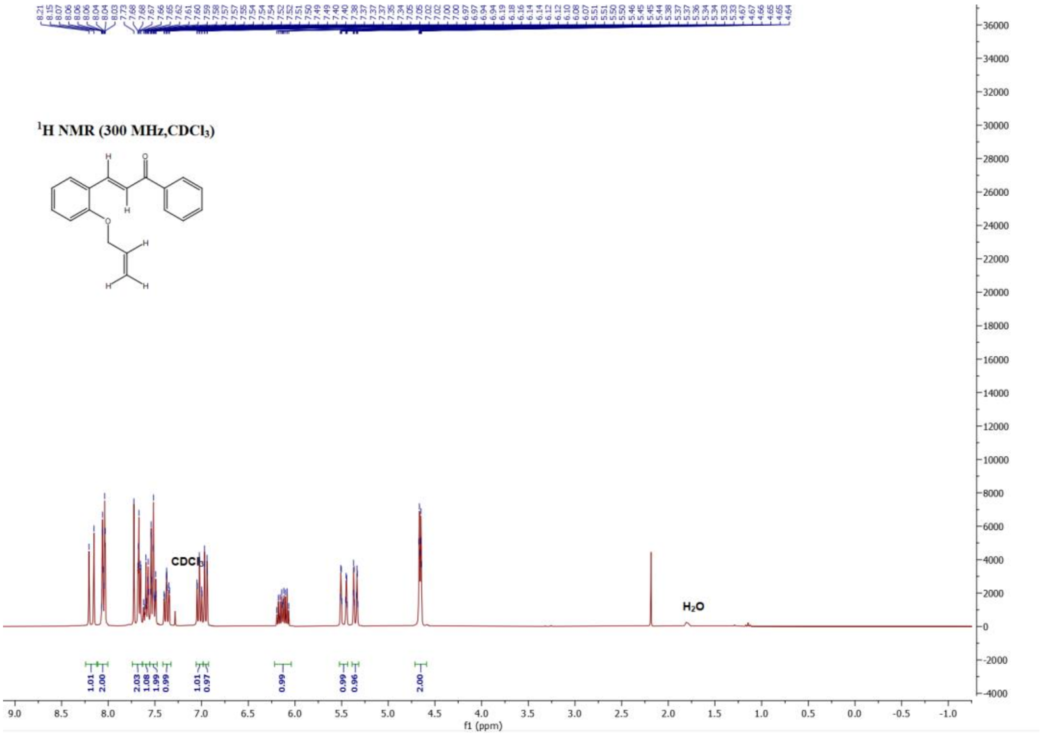

¹³C NMR for 3a

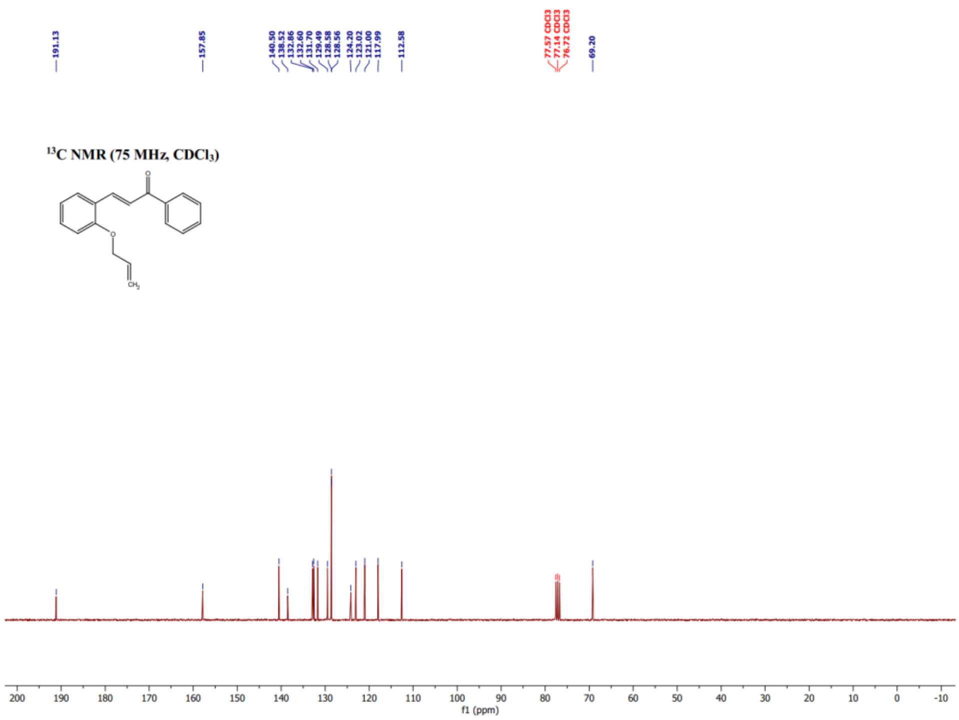

^{1}H NMR for 3b

(*E*)-2-(3-(2-(allyloxy)phenyl)acryloyl)cyclohexan-1-one

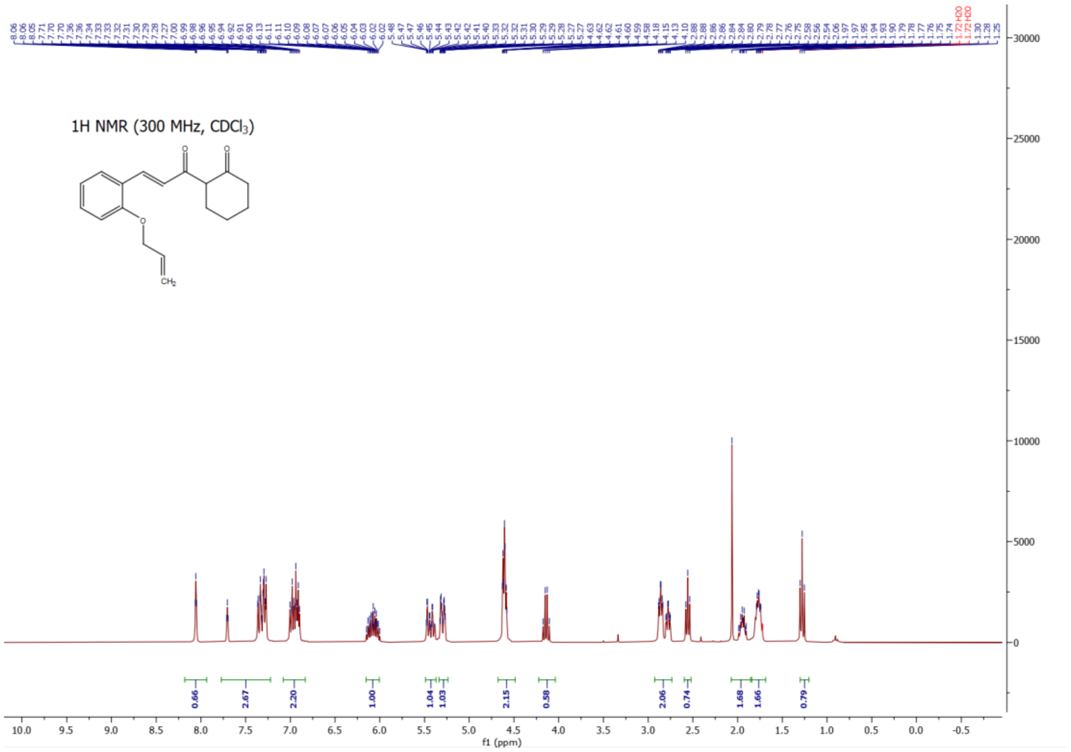

^{13}C NMR for 3b

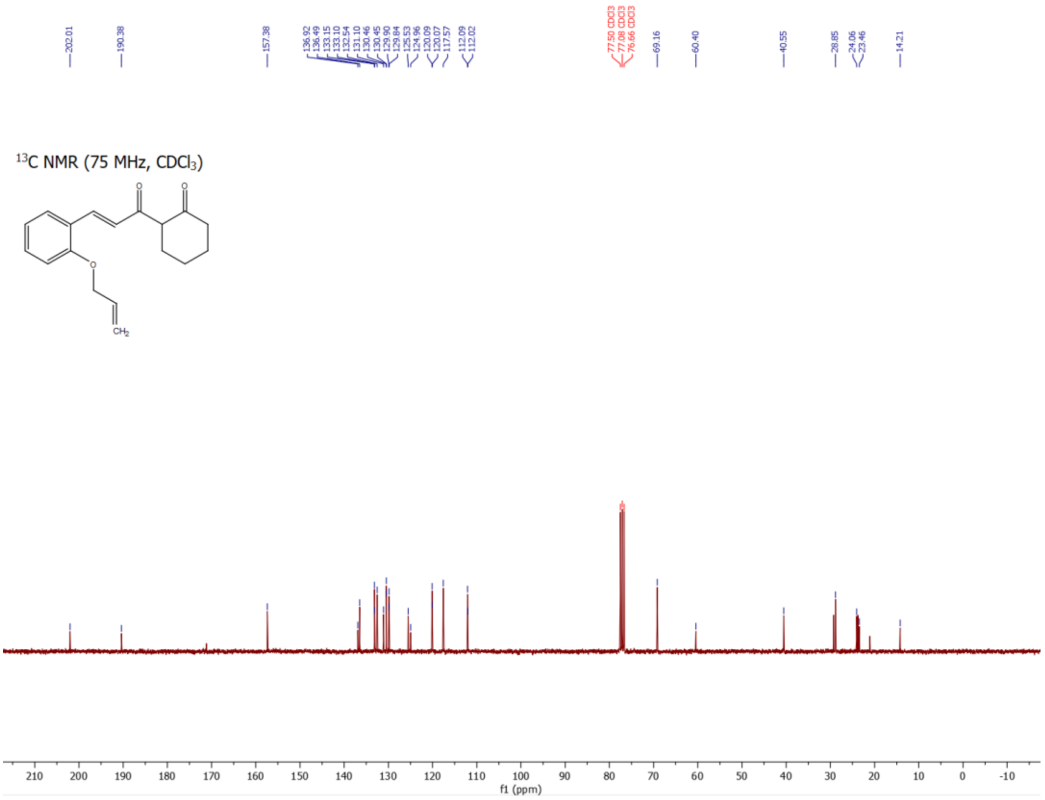

^{1}H NMR for 3c

(*E*)-1-(naphthalen-1-yl)-3-(2-nitrophenyl)prop-2-en-1-one

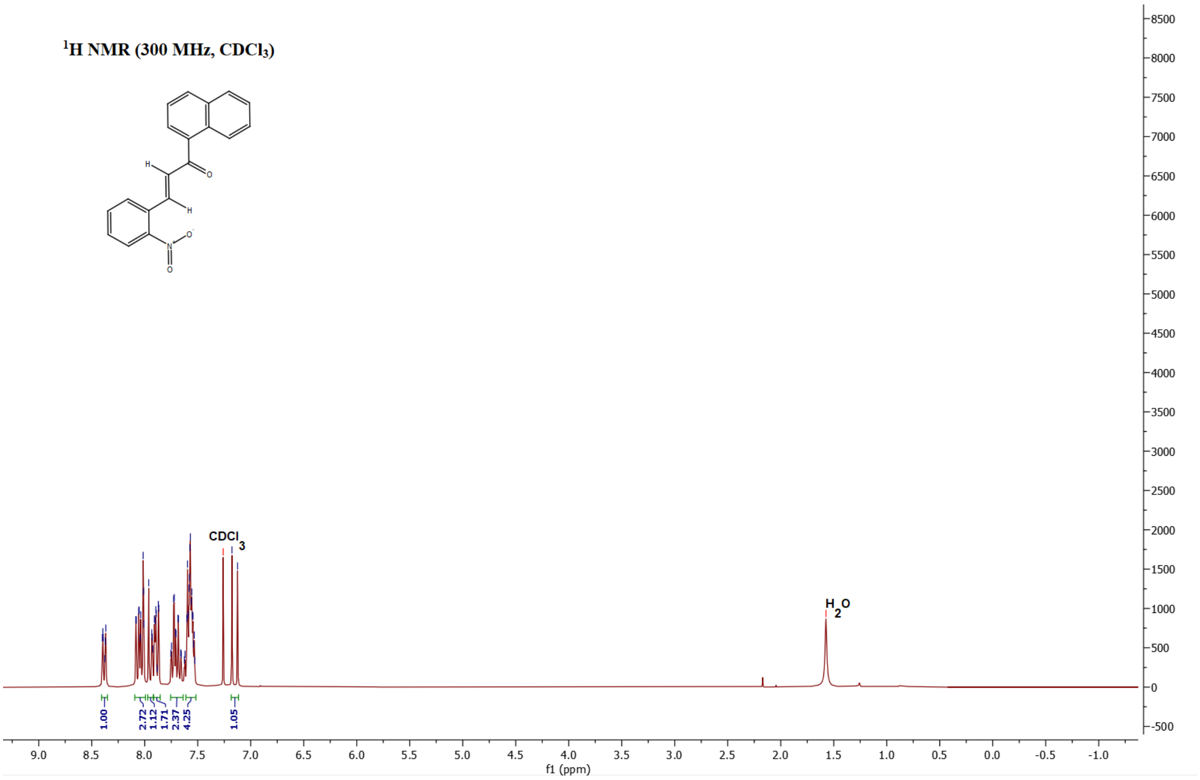

^{13}C NMR for 3c

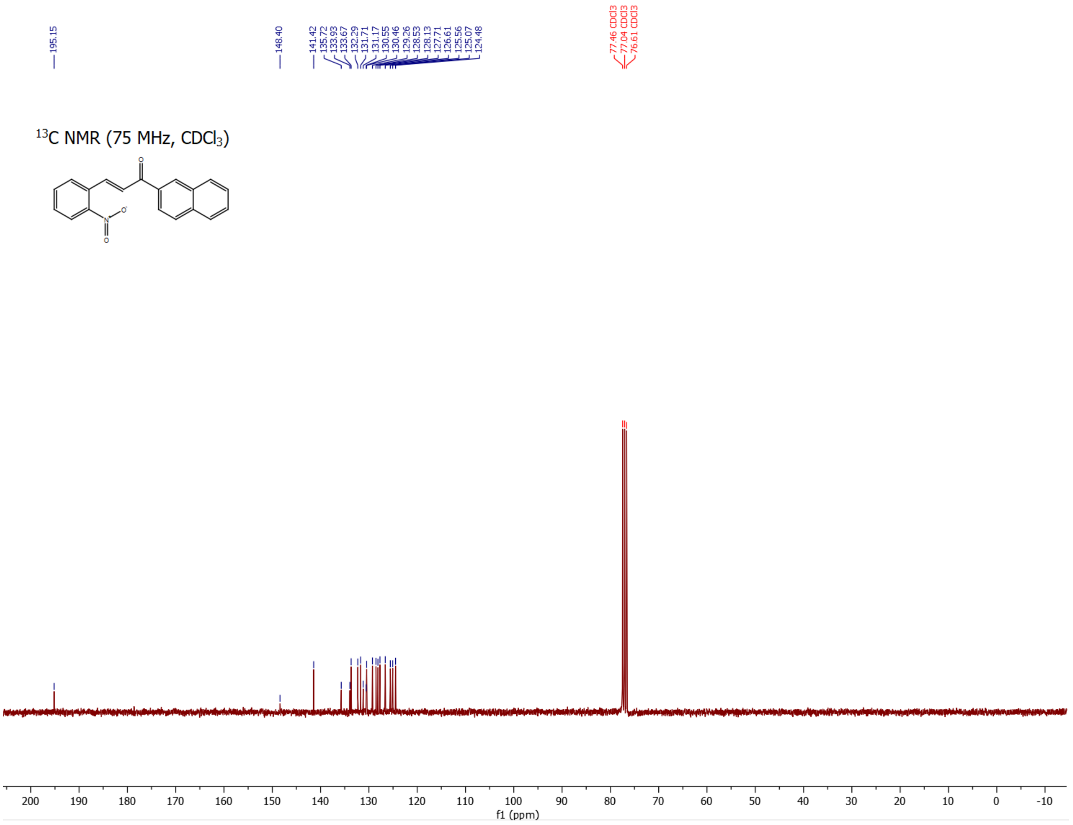

^{1}H NMR for 3d

(*E*)-3-(2-(allyloxy)phenyl)-1-(4-aminophenyl)prop-2-en-1-one

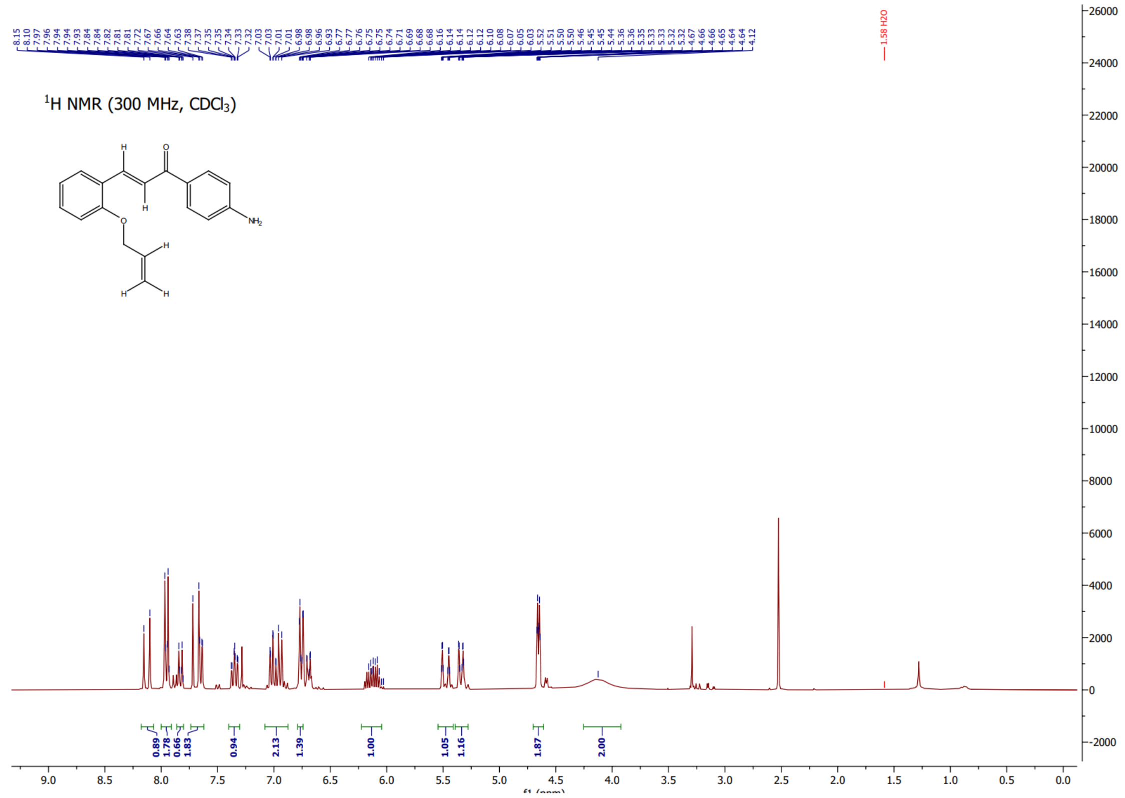

^{13}C NMR for 3d

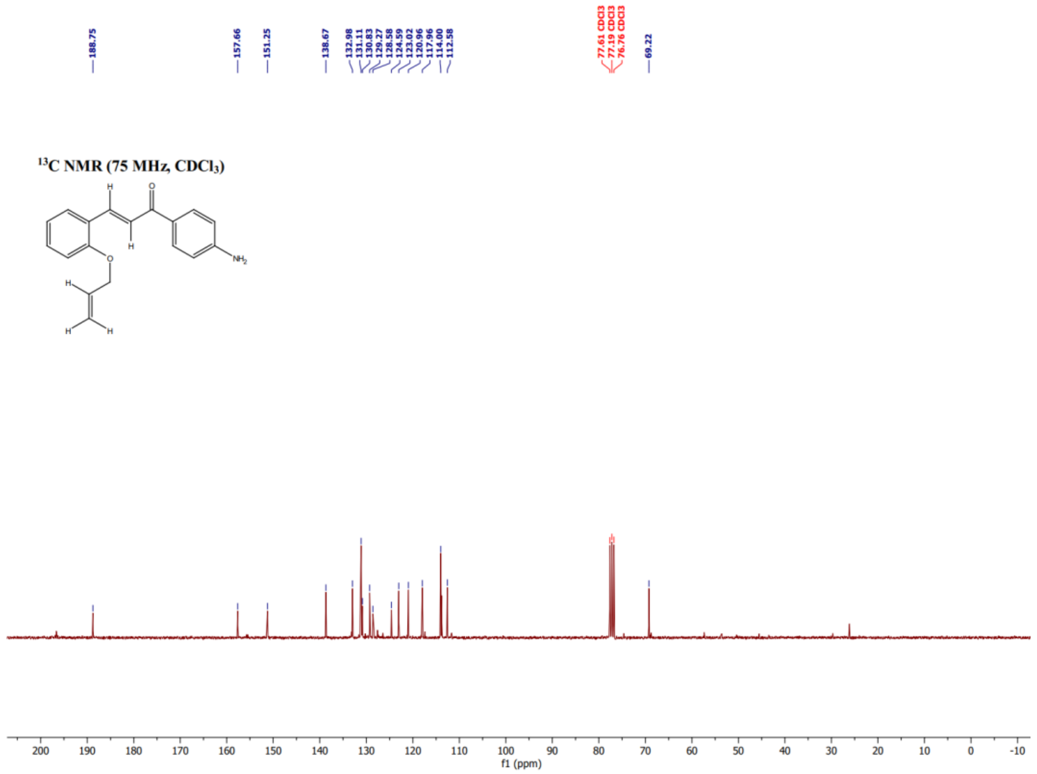

^{1}H NMR for 3e

(*E*)-3-(2-(allyloxy)phenyl)-1-(naphthalen-2-yl)prop-2-en-1-one

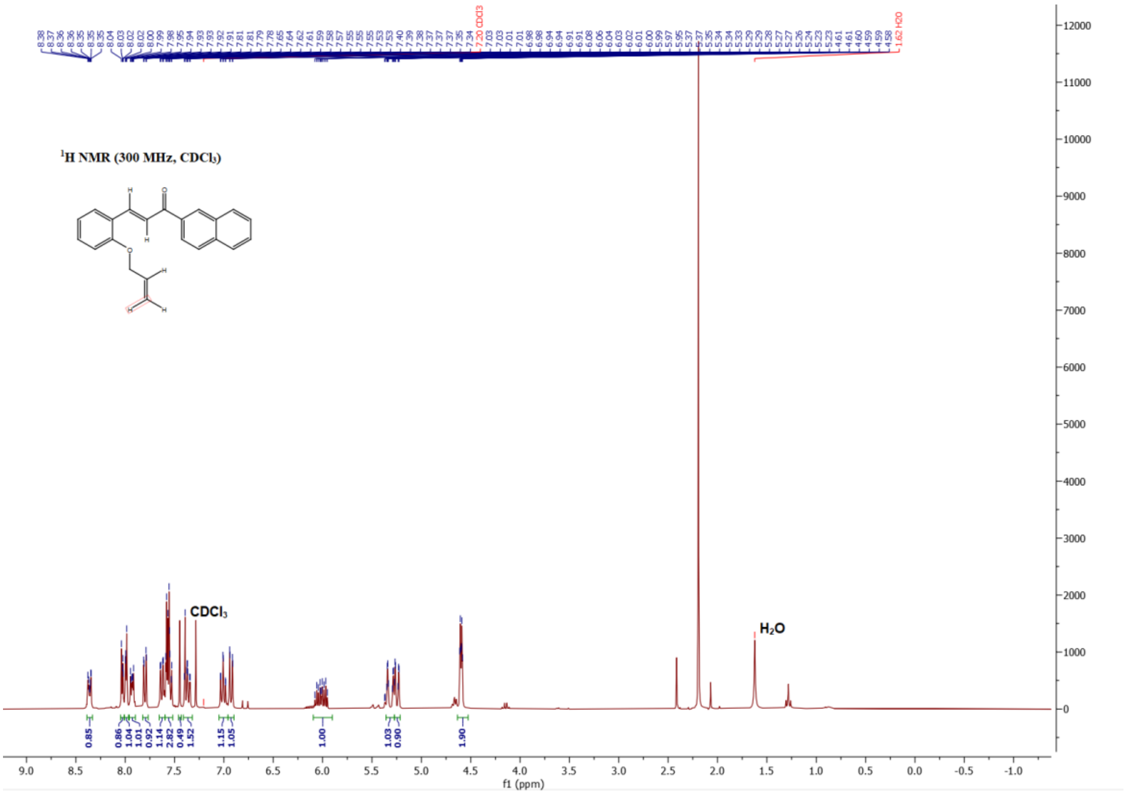

^{13}C NMR for 3e

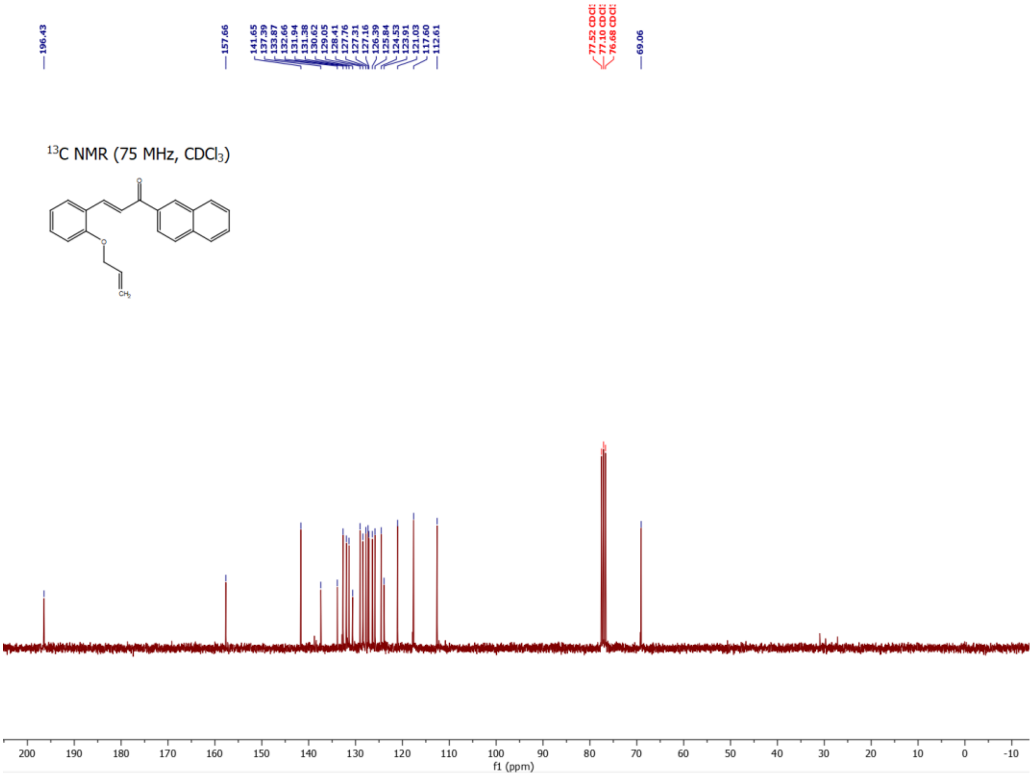

^{1}H NMR for 3f

(*E*)-2-(3-(naphthalen-2-yl)-3-oxoprop-1-en-1-yl)phenyl benzenesulfonate

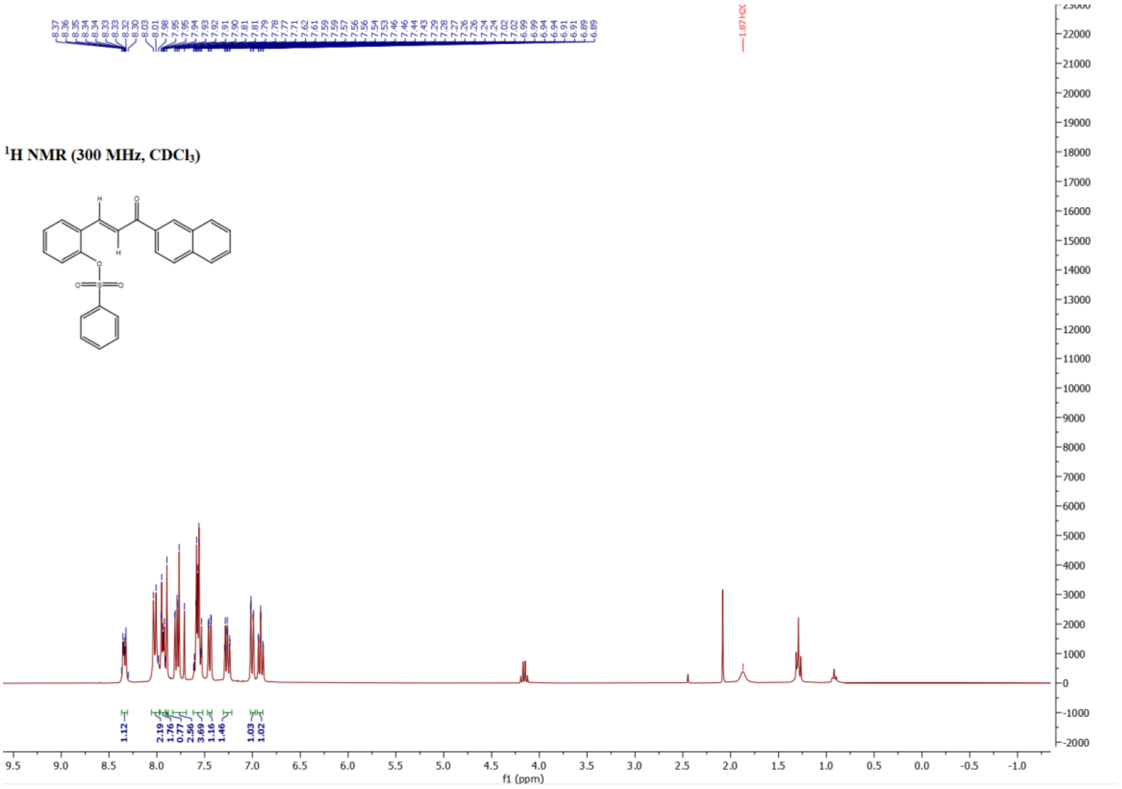

^{13}C NMR for 3f

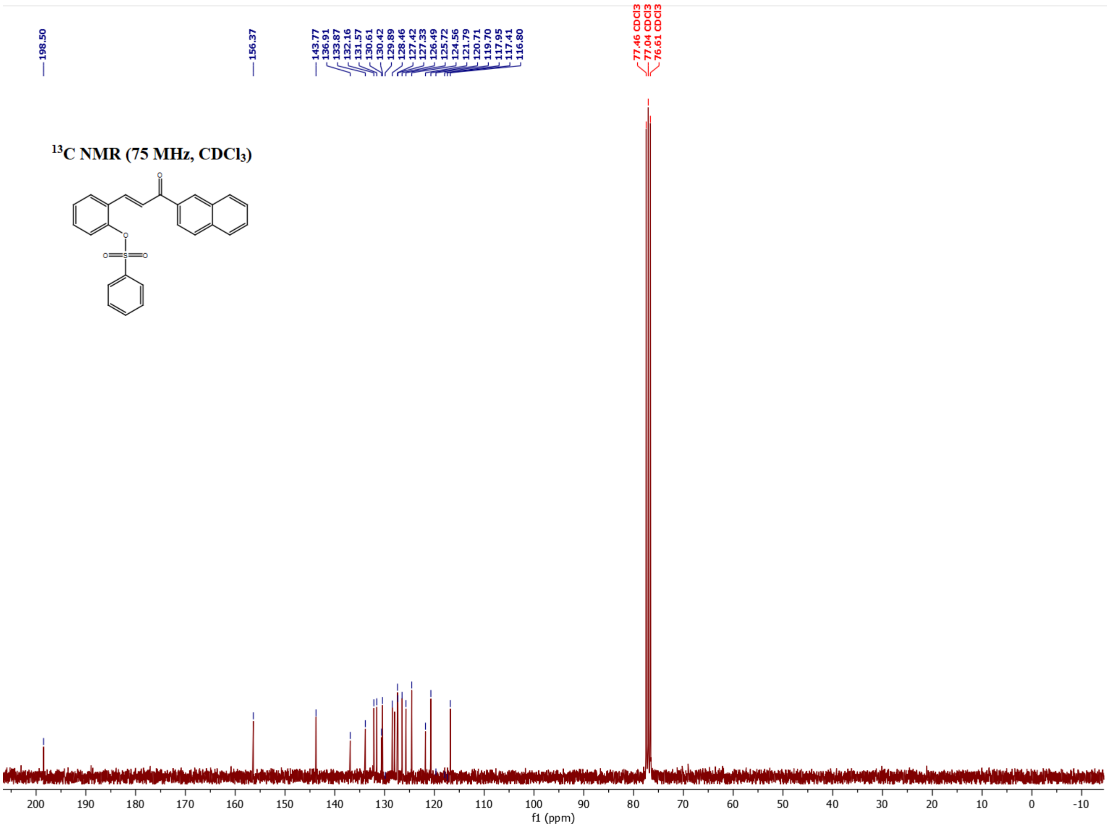

^{1}H NMR for 3g

(*E*)-3-(2-(allyloxy)-5-methoxyphenyl)-1-(naphthalen-2-yl)prop-2-en-1-one

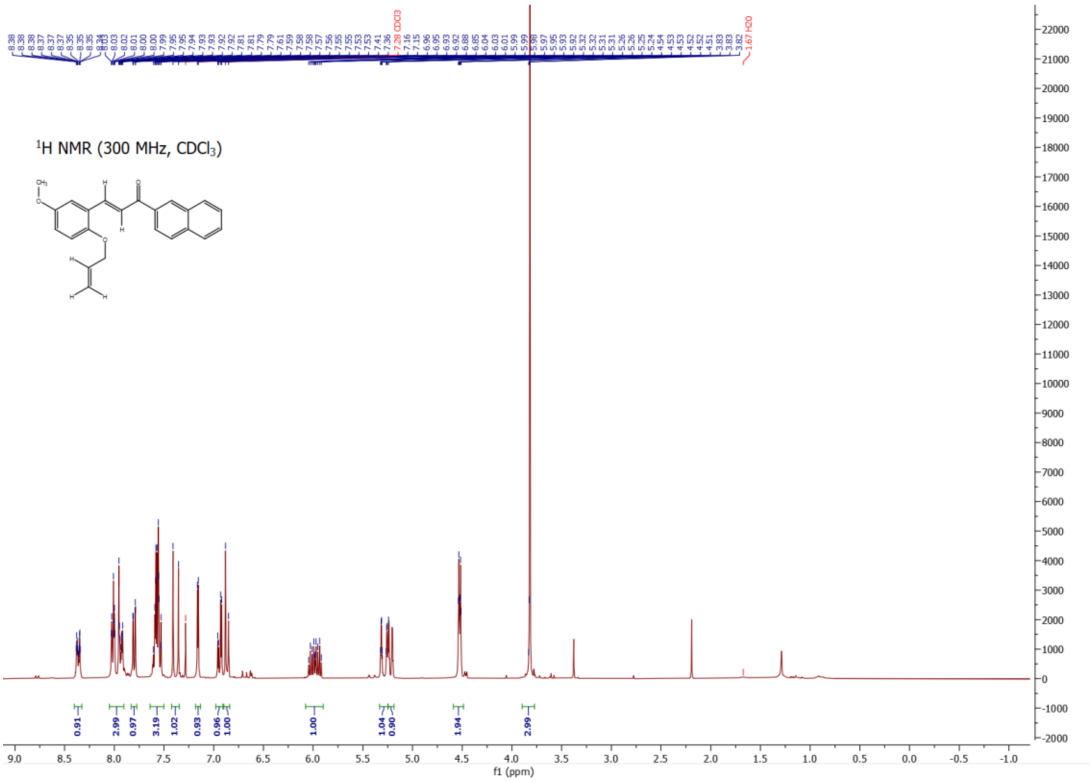

^{13}C NMR for 3g

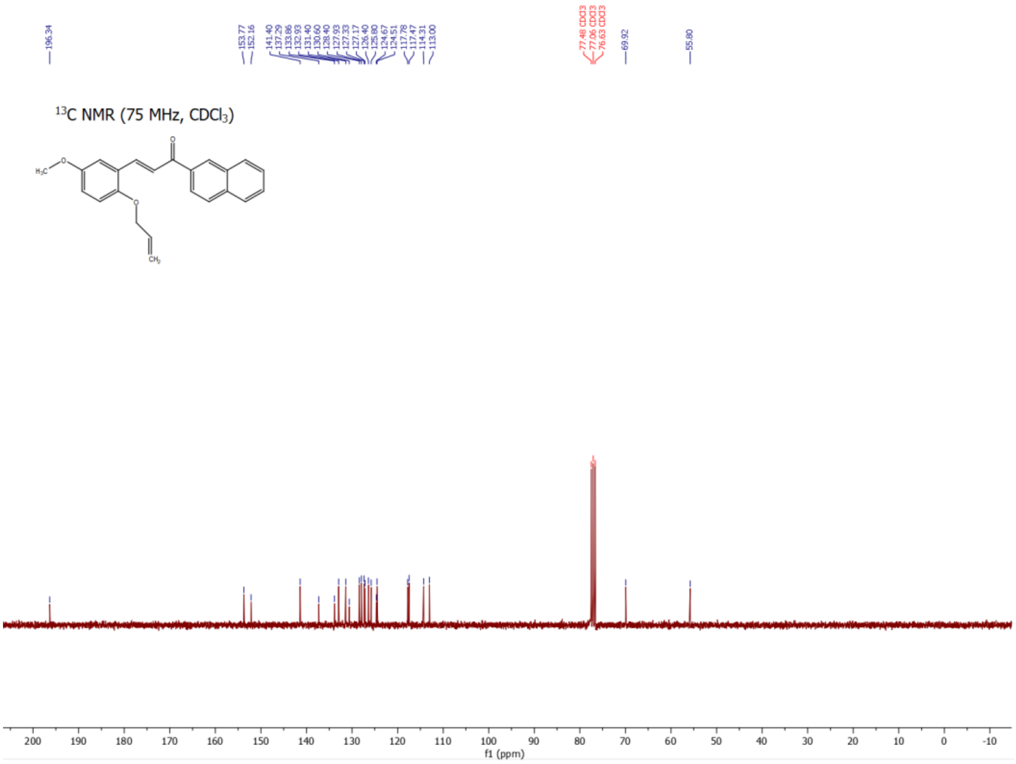

^{1}H NMR for 3h

(*E*)-1-(4-aminophenyl)-3-(2-((3-methylbut-2-en-1-yl)oxy)phenyl)prop-2-en-1-one

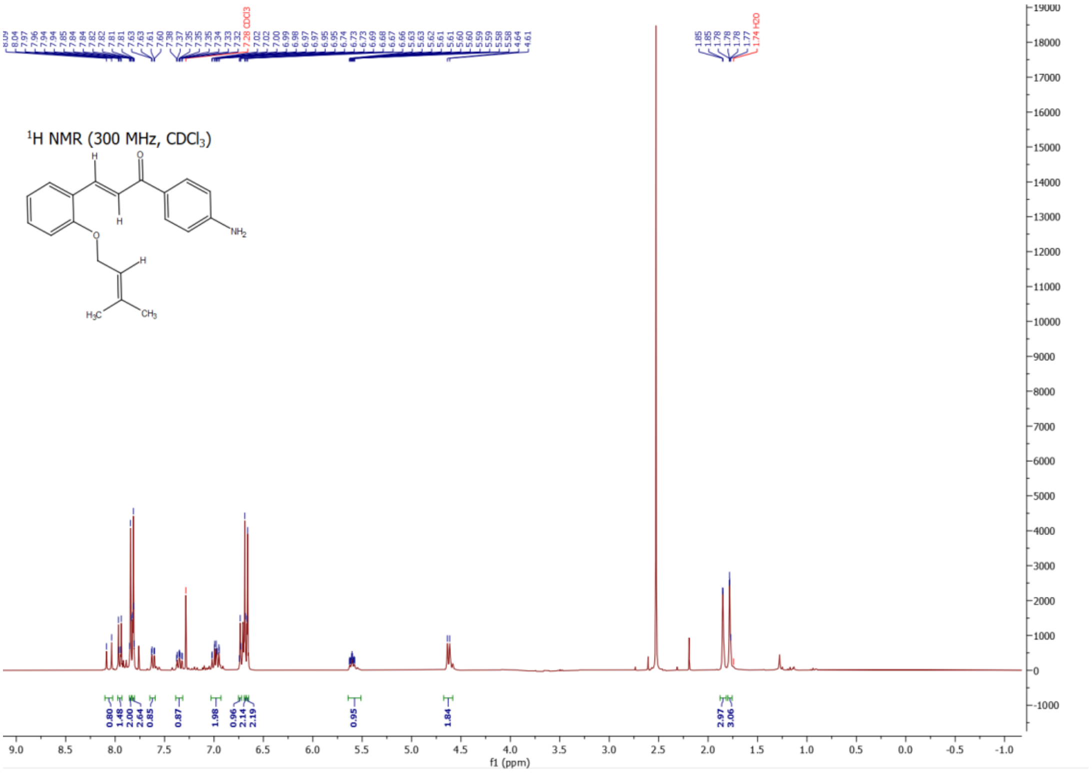

^{13}C NMR for 3h

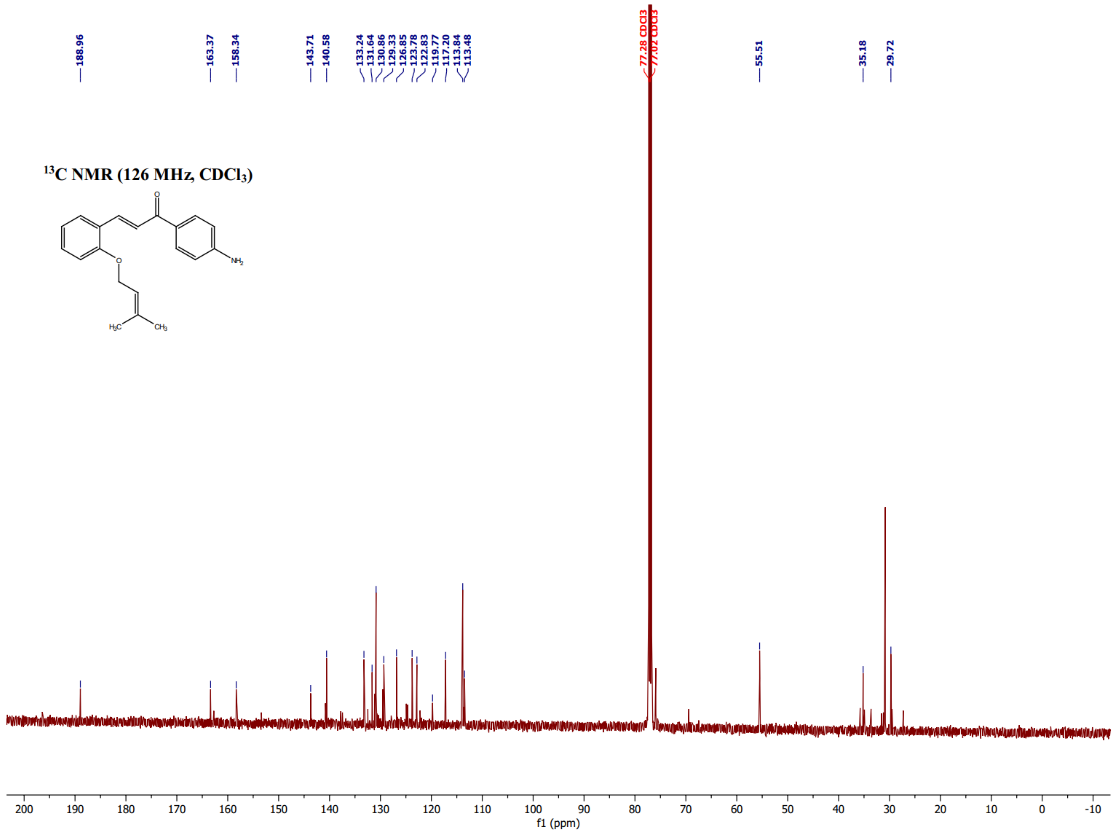

^{1}H NMR for 3i

(*E*)-3-(2-(allyloxy)-3-(tert-butyl)phenyl)-1-(4-methoxyphenyl)prop-2-en-1-one

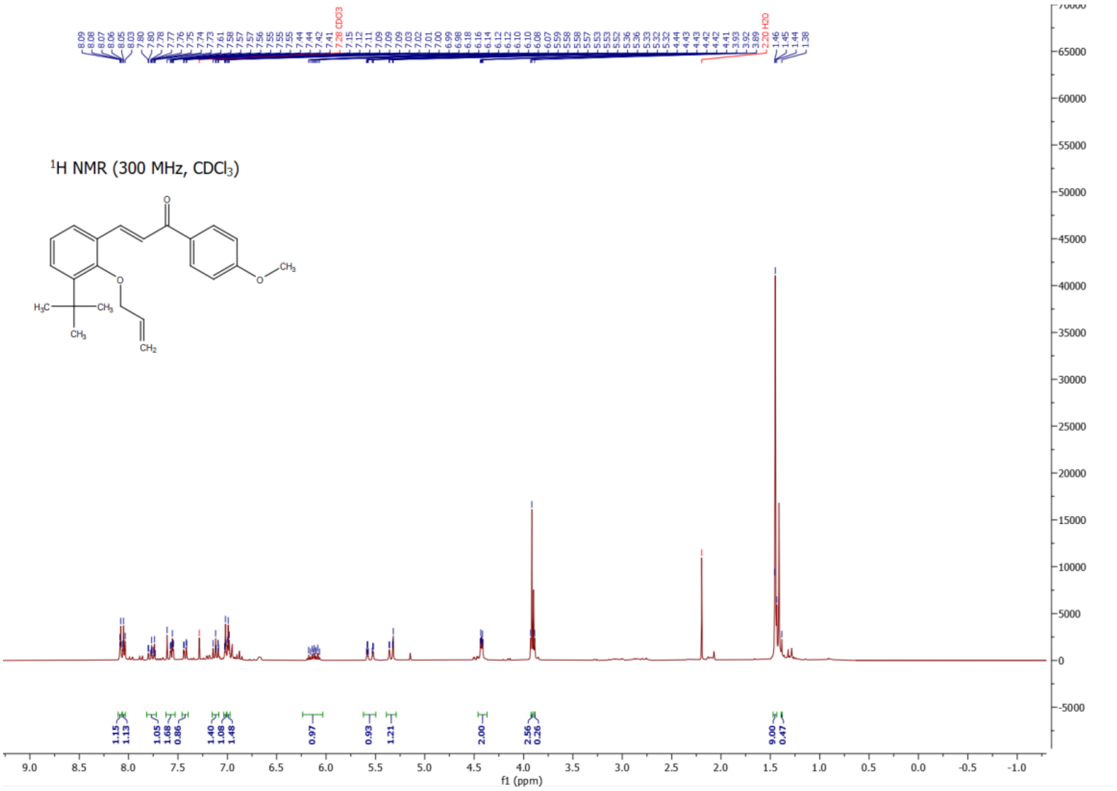

^{13}C NMR for 3i

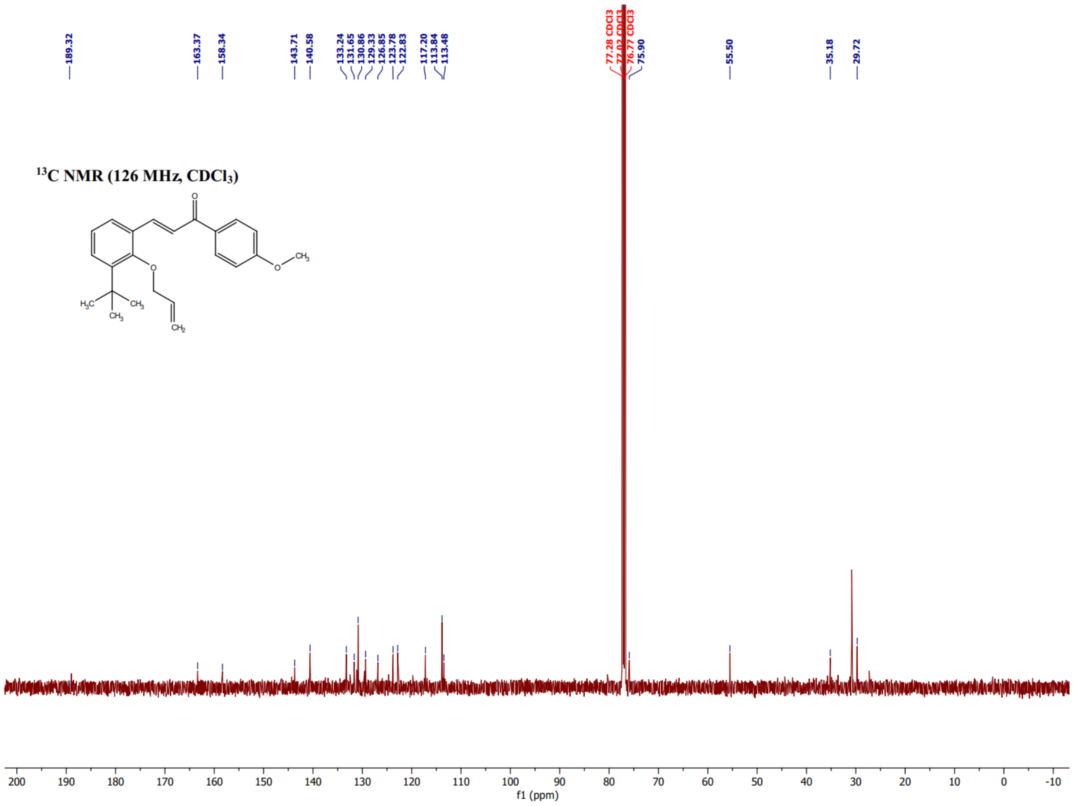

^{1}H NMR for 3j

(*E*)-3-(2-(allyloxy)-3-(tert-butyl)phenyl)-1-phenylprop-2-en-1-one

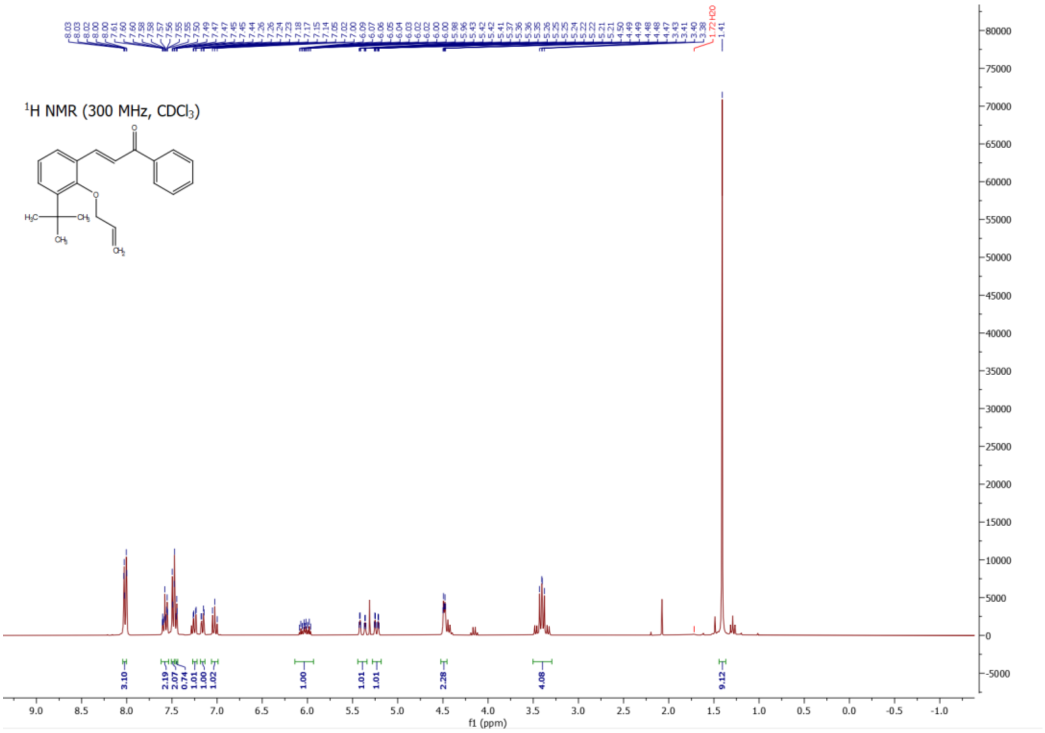

^{13}C NMR for 3j

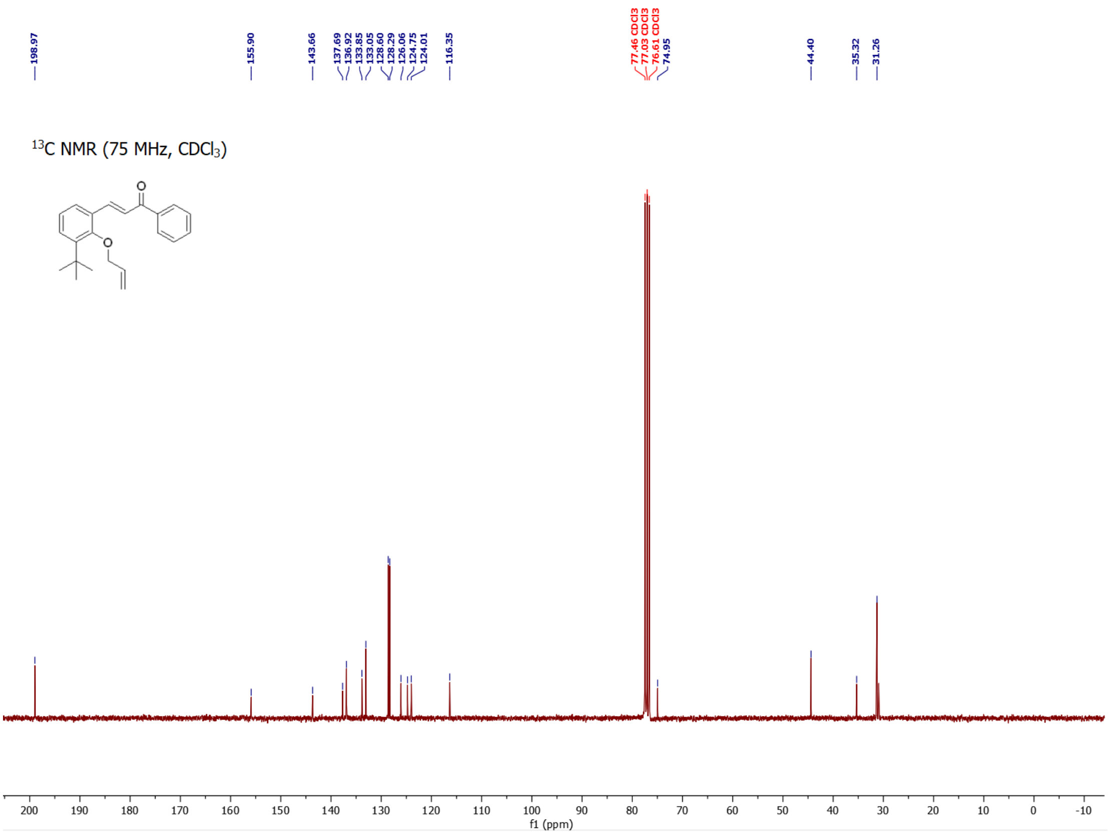

^{1}H NMR for 3k

(*E*)-3-(2-(allyloxy)-3,5-dibromophenyl)-1-phenylprop-2-en-1-one

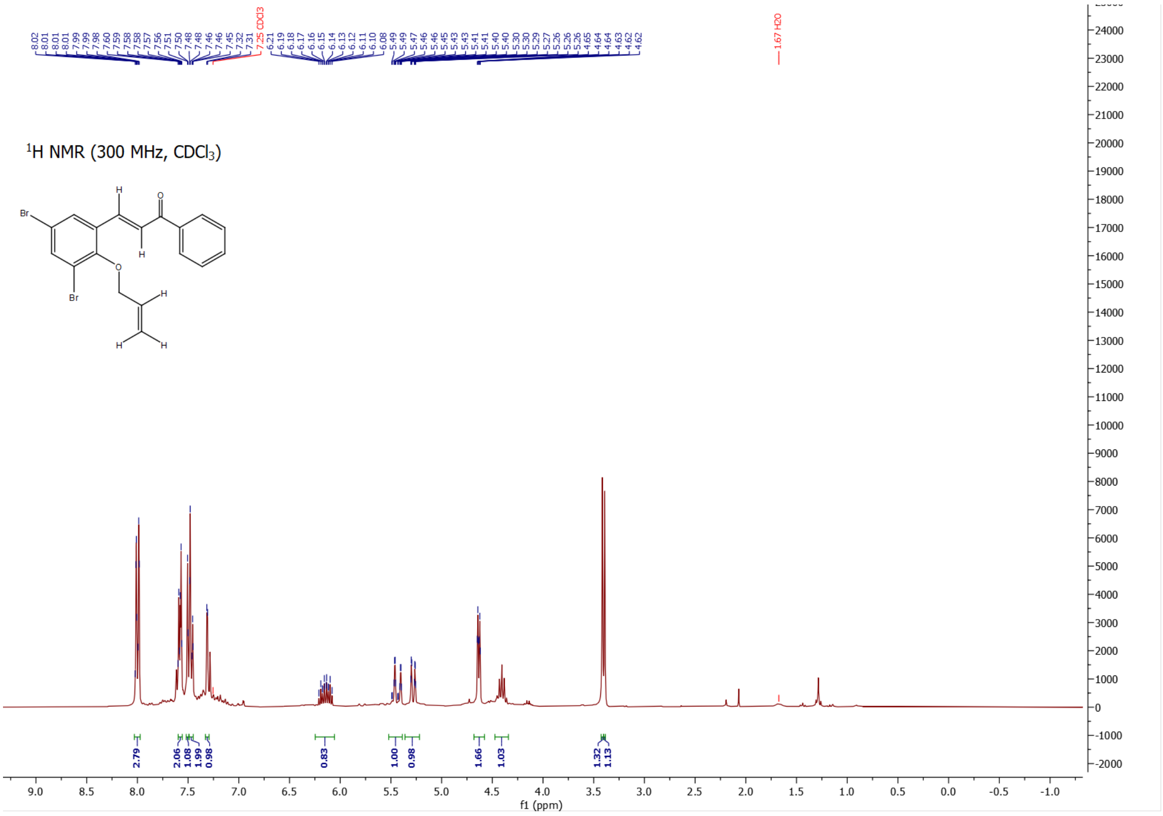

^{13}C NMR for 3k

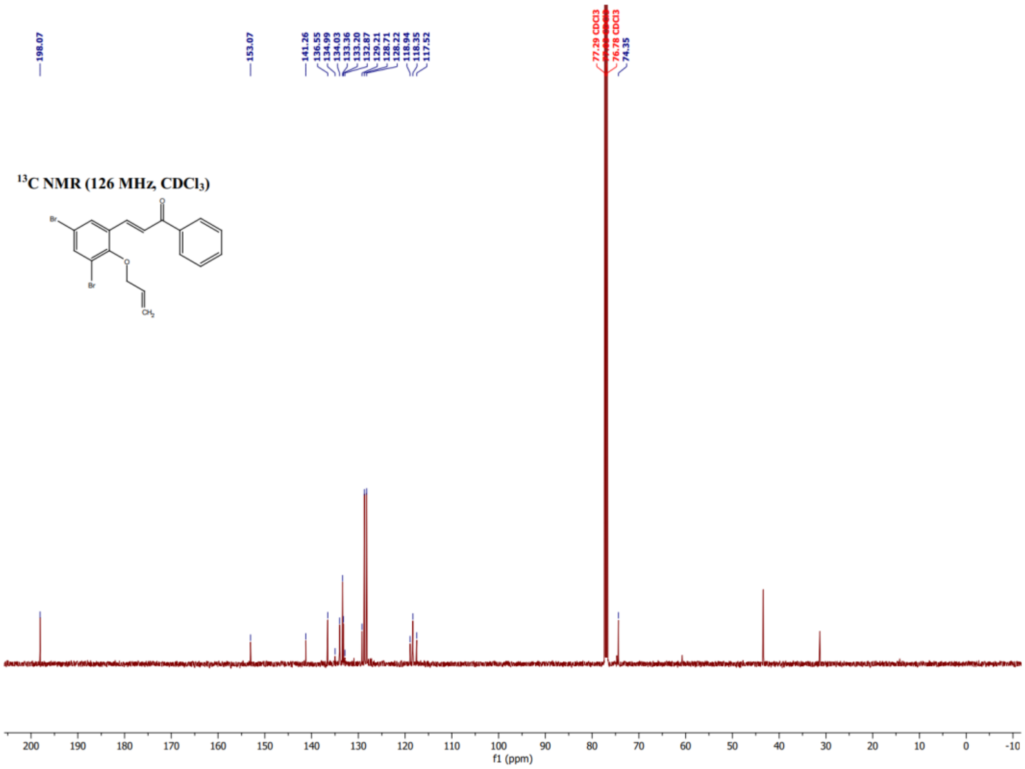

^{1}H NMR for 3l

(*E*)-3-(2-(allyloxy)-3,5-dichlorophenyl)-1-(4-aminophenyl)prop-2-en-1-one

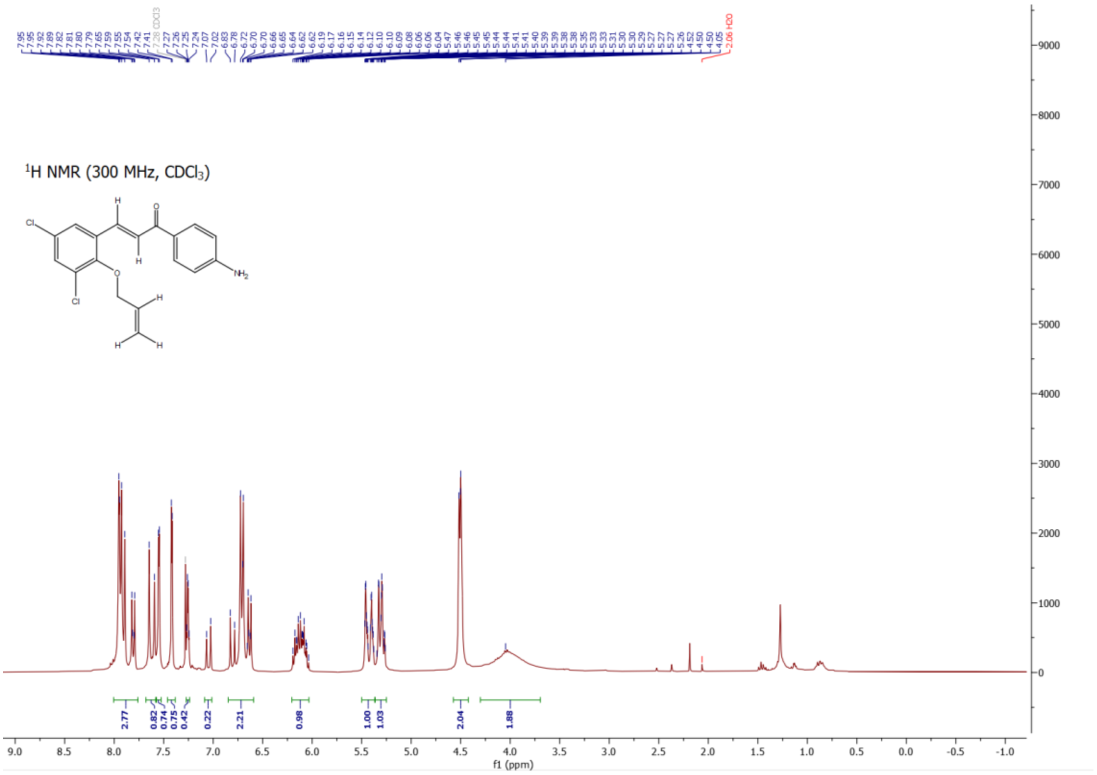

^{13}C NMR for 3l

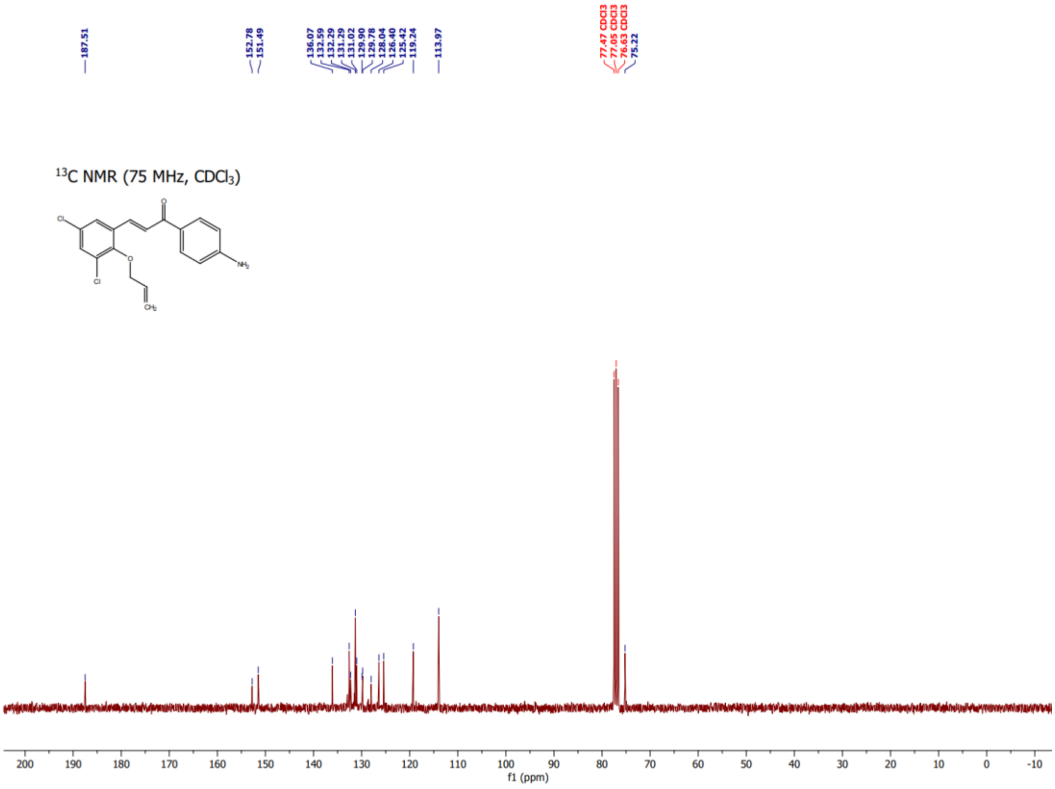

Supplement: Supplementary file 1 [file antioxidants-11-01846-s001.zip › Supplementary Materials-1-Final.pdf]
