# Supplementary material for: Novel Cinnamaldehyde Derivatives Inhibit Peripheral Nerve Degeneration by Targeting Schwann Cells
Source: Antioxidants (Basel). 2022 Sep 20;11(10):1846. doi: 10.3390/antiox11101846 (PMC9598575; doi:10.3390/antiox11101846)
Supplement: Supplementary file 1 [file antioxidants-11-01846-s001.zip › Supplementary Materials-2-Final.pdf]

Entry	Concentration (μM)		
	IC_{50}	GI_{50}	EC_{50}
CAH	NA	24.8 ± 1.5	NA
CL	NA	11.1 ± 0.1	NA
3a	82.7 ± 1.2	12.0 ± 4.0	91.2 ± 1.6
3b	NA	63.7 ± 4.2	NA
3c	NA	4.0 ± 0.3	NA
3d	NA	10.3 ± 1.7	NA
3e	NA	24.3 ± 1.1	NA
3f	79.7 ± 0.9	7.5 ± 1.0	97.8 ± 0.2
3g	68.6 ± 5.8	3.7 ± 0.6	NA
3h	95.5 ± 0.2	12.6 ± 1.6	NA
3i	NA	NA	NA
3j	NA	NA	NA
3k	65.6 ± 3.0	9.1 ± 3.0	NA
3l	NA	6.5 ± 4.7	NA

* NA: not available

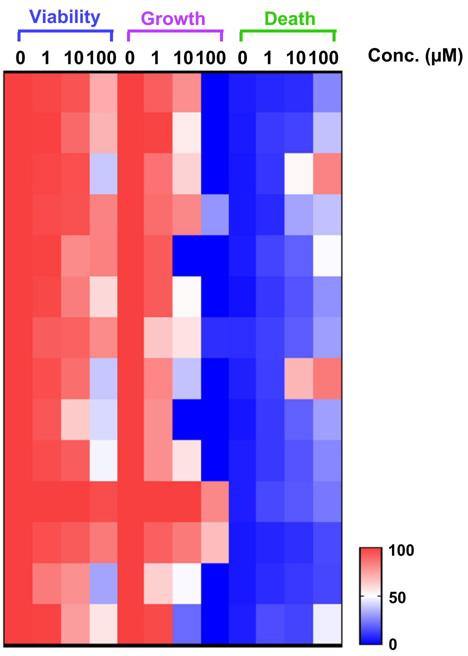

Figure S1. Therapeutic indices of CAH derivatives in dedifferentiated SW10 cells. The table (left panel) designated the corresponding concentration of half maximal inhibitory concentration (IC_{50}), half-maximal growth inhibition (GI_{50}), and half maximal effective concentration (EC_{50}) in the SW10 cells with **3a-3l** treatment. The heatmap (right panel) represented the concentration-dependent change of cell viability (%), growth inhibition (%), and cell death (%) of SW10 cells treated with **3a-3l**.

A

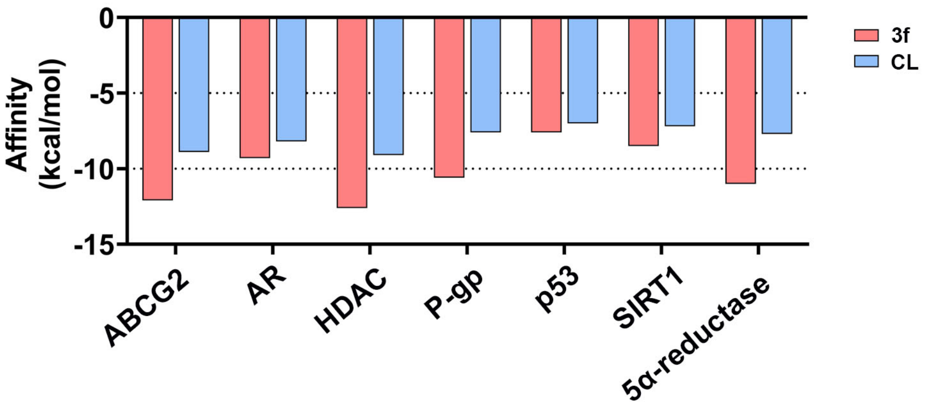

B

Protein	RCSB ID	Affinity (kcal/mol)	
		3f	CL
ABCG2	6VXI	-12.1	-8.9
AR	1E3G	-9.3	-8.2
HDAC	1C3R	-12.6	-9.1
P-gp	6C0V	-10.6	-7.6
p53	2OCJ	-7.6	-7.0
SIRT1	5BTR	-8.5	-7.2
5α-reductase	7C83	-11.0	-7.7

Figure S2. Bindig affinity between protein targets of chalcone (CL) and **3f**. (A) Binding free energy of **3f** and CL with each target was shown as red and blue bars, respectively. (B) Binding potency was compared between **3f** and CL. AR, adrogen receptor; P-gp, P-glycoprotein.

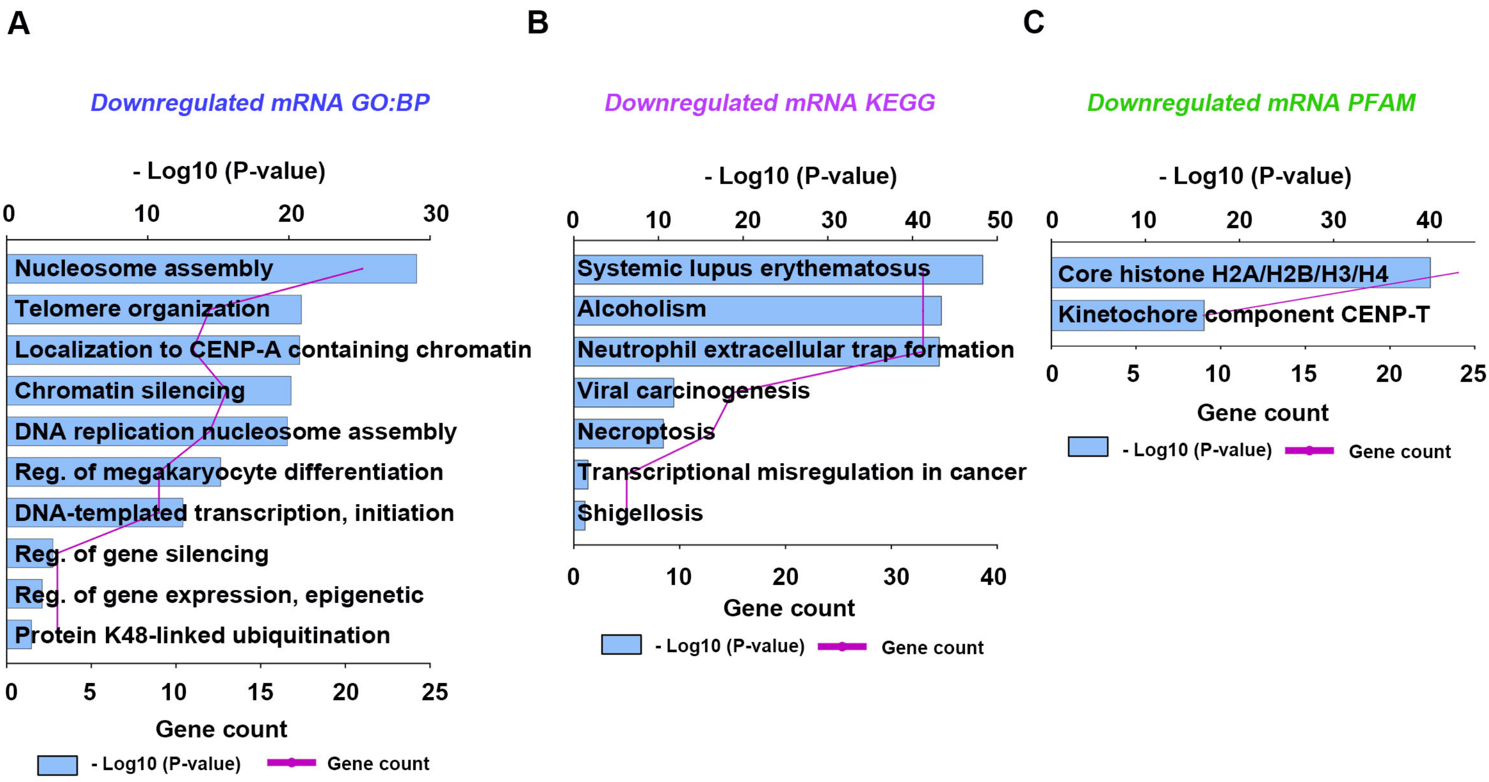

Figure S3. Gene ontology (GO), Kyoto Encyclopedia of Genes and Genomes (KEGG), and Protein Family (PFAM) assays using downregulated differentially expressed genes (DEGs). The GO:BP, KEGG pathway, and PFAM enrichments were shown in (A), (B), and (C), respectively. The upper x-axis of each graph indicated GO:BP term, KEGG pathway, and PFAM, respectively. The lower x-axis showed the number of genes involved in the corresponding term. BP: Biological process.

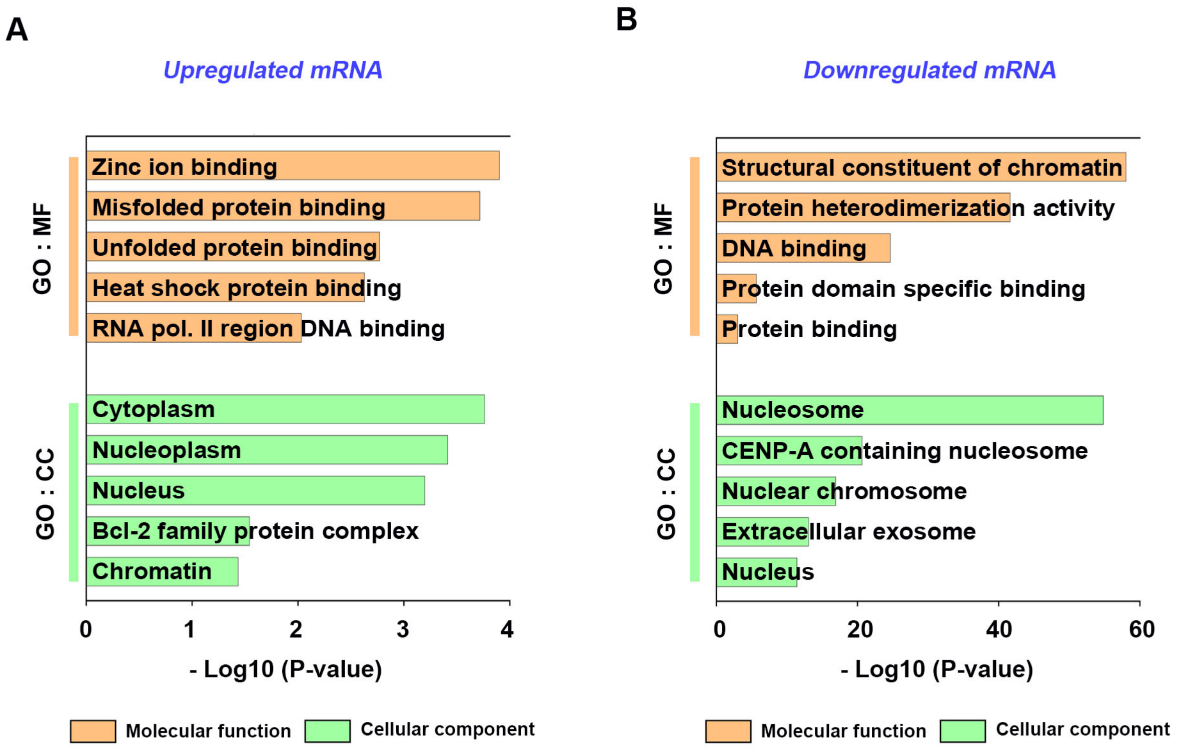

Figure S4. GO molecular function (MF) and cellular component (CC) analysis using upregulated and downregulated DEGs. The GO categories contained the top 5 terms of GO:MF or GO:CC were indicated in (A) and (B), respectively. Top 5 enrichment scores were obtained from the DAVID database.

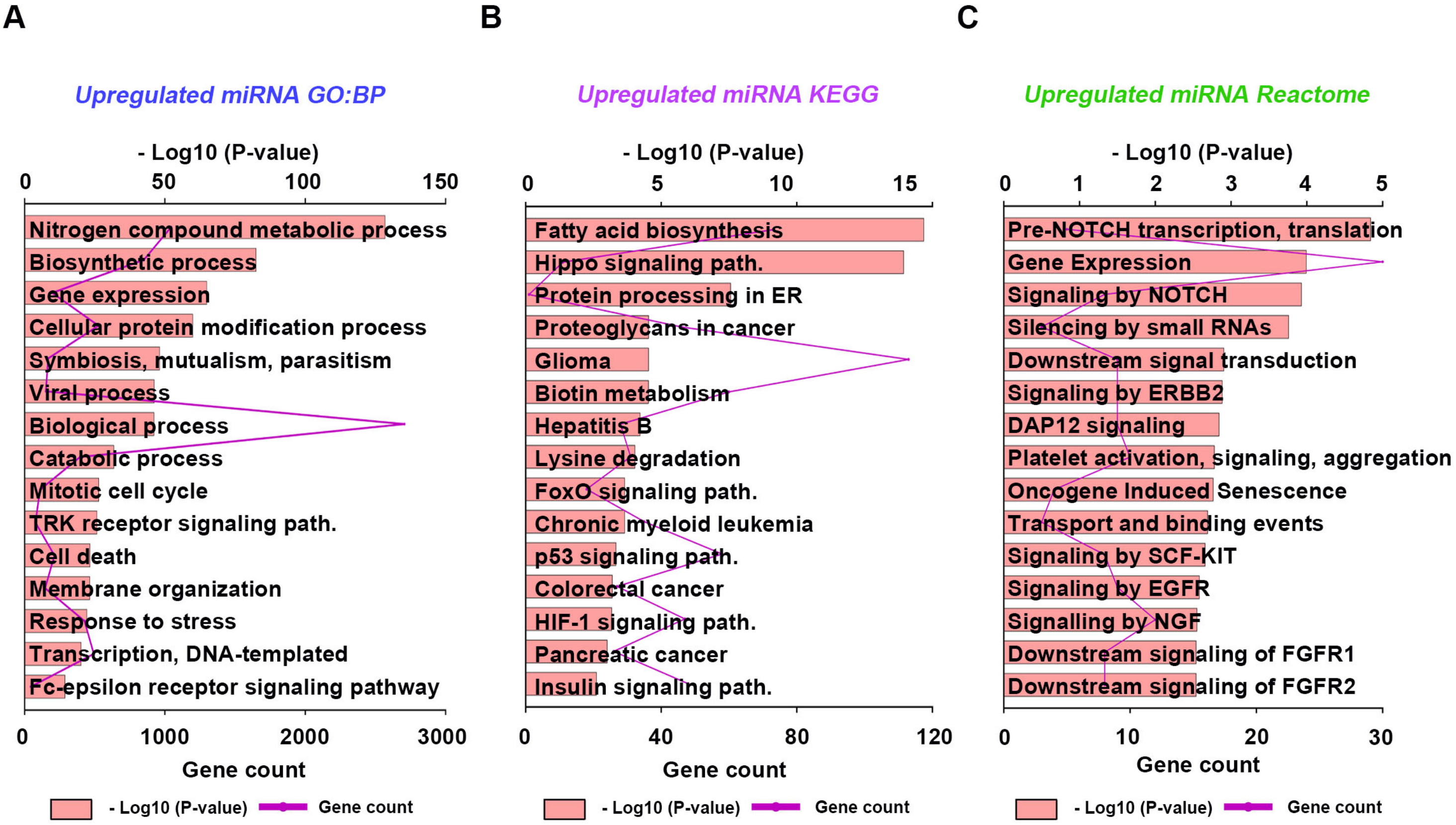

Figure S5. GO terms, KEGG pathway, and Reactome assays using upregulated differentially expressed miRNAs (DE miRNAs). The GO:BP, KEGG pathway, and Reactome enrichments were shown in (A), (B), and (C), respectively.The upper x-axis of each graph indicated GO:BP terms, KEGG pathway, and Reactome respectively. The lower x-axis implied the number of genes involved in each term.

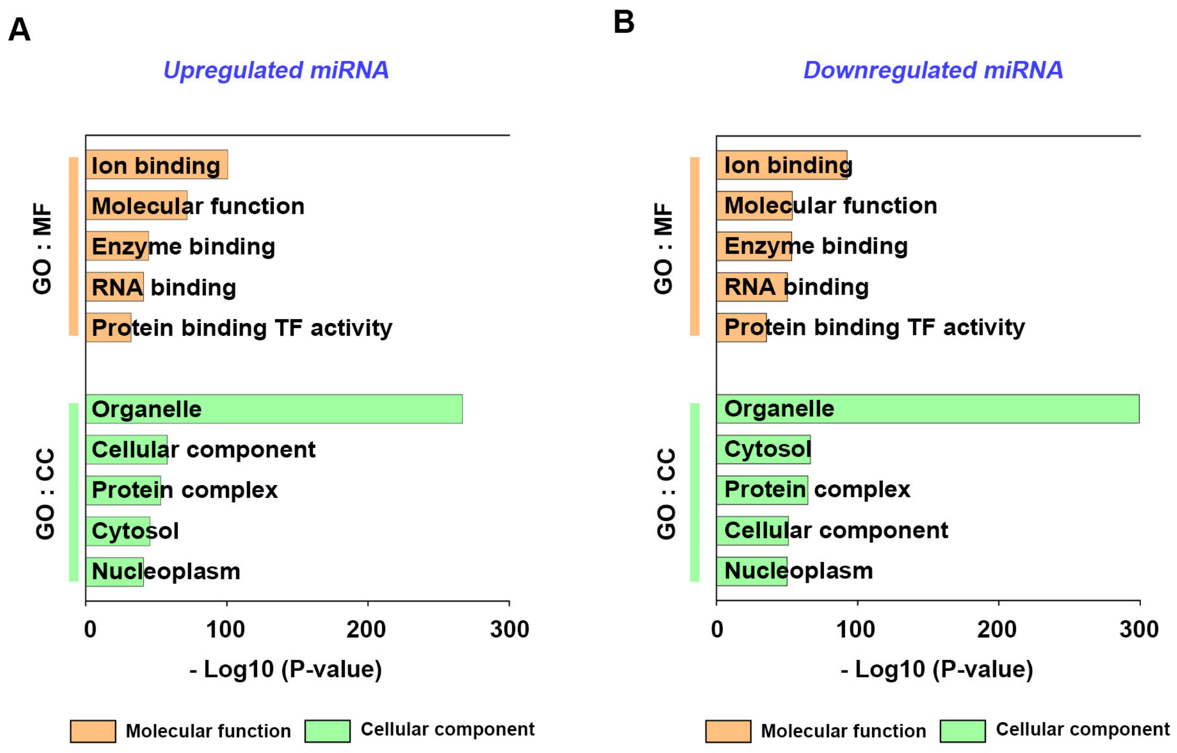

Figure S6. GO molecular function (MF) and cellular component (CC) analysis using upregulated and downregulated DE miRNAs. The GO categories contained the top 5 terms of GO:MF or GO:CC were indicated in (A) and (B), respectively. Top 5 enrichment scores were obtained from the DAVID database.

Table S1. Primer sequences used in qPCR validation

Gene	Forward (5'-3')	Reverse (5'-3')	bp
TSPYL2	GCAAGTTCATCCAGATGCGA	GTGGTTGAGGAATGCTTTGAC	95
GDF9	GAGTGTGAGCTCCATGACTTT	CCCTTTACAGTATCGAGGGTTG	99
SESN2	CAGCCTATAGCCTCACCTAC	TATCTGATGCCAAAGACGCA	109
CRYAB	GTCAACCTGGATGTGAAGCACTT	TTCATCCTGGCGCTCTTCAT	102
HSPA1B	CCATTGAGGAGGTGGATTAGGG	ATGACATAGGAAAACAGCAGCA	100
NR4A2	GGGATGGTCAAAGAAGTGGTTCG	GGTCATAGCCGGGTTGGAGTC	162
DHRS2	GCCCTACATGGAGAACAGGA	AGCTCCAATGCCAGTGTTCT	138
GAPDH	GGAAGGTGAAGGTCGGAGTCA	GTCATTGATGGCAACAATATCCACT	101
hsa-miR-1246	AATGGATTTTTGGAGCAGG		
hsa-miR-147b-3p	GTGTGCGGAAATGCTTCTGCT		
hsa-miR-1303	TTTAGAGACGGGGTCTTGCTCT		
hsa-miR-320d	AAAAGCTGGGTTGAGAGGA		
hsa-miR-193a-3p	AACTGGCCTACAAAGTCCCAGT	QuantMir Universal Primer (System Biosciences)	
hsa-miR-23a-3p	ATCACATTGCCAGGGATTTCC		
hsa-miR-16-2-3p	CCAATATTACTGTGCTGCTTTA		
hsa-miR-7-5p	TGGAAGACTAGTGATTTTGTTGTT		
hsa-miR-425-5p	AATGACACGATCACTCCCGTTGA		
hsa-miR-155-5p	TTAATGCTAATCGTGATAGGGGTT		
human U6	CGCAAGGATGACACGCAAATTC		

Table S2. Molecular docking between TRPA1 and CAH derivatives

Protein (RCSB ID): TRPA1 (6X2J)			
Ligand	Affinity (kcal/mol)	Ligand	Affinity (kcal/mol)
3a	-7.4	3g	-8.4
3b	-7.2	3h	-7.5
3c	-8.8	3i	-7.2
3d	-7.4	3j	-7.3
3e	-9	3k	-6.6
3f	-9.6	3l	-7.3
